# Functional and structural connectivity of thalamic subnuclei in major depressive disorder at 7 Tesla

**DOI:** 10.1111/pcn.70048

**Published:** 2026-03-11

**Authors:** Weijian Liu, Shu Liu, Jurjen Heij, Laurens A. van de Mortel, Luka Liebrand, Matthan Caan, Wietske van der Zwaag, Dick J. Veltman, Lin Lu, Moji Aghajani, Guido van Wingen

**Affiliations:** ^1^ Department of Psychiatry Amsterdam UMC Location University of Amsterdam Amsterdam The Netherlands; ^2^ Amsterdam Neuroscience Amsterdam The Netherlands; ^3^ Danish Research Centre for Magnetic Resonance, Department of Radiology and Nuclear Medicine Copenhagen University Hospital Amager and Hvidovre Hvidovre Denmark; ^4^ Peking University Sixth Hospital, Peking University Institute of Mental Health, NHC Key Laboratory of Mental Health (Peking University), National Clinical Research Center for Mental Disorders (Peking University Sixth Hospital), Chinese Academy of Medical Sciences Research Unit (No. 2018RU006) Peking University Beijing China; ^5^ Key Laboratory of Genetic Evolution & Animal Models, National Research Facility for Phenotypic & Genetic Analysis of Model Animals (Primate Facility), National Resource Center for Non‐Human Primates, Kunming Institute of Zoology Chinese Academy of Sciences Kunming China; ^6^ Spinoza Centre for Neuroimaging KNAW Amsterdam The Netherlands; ^7^ Netherlands Institute for Neuroscience KNAW Amsterdam The Netherlands; ^8^ Department of Radiation Oncology Amsterdam UMC Location Vrije Universiteit Amsterdam Amsterdam The Netherlands; ^9^ Department of Biomedical Engineering & Physics Amsterdam UMC location University of Amsterdam Amsterdam The Netherlands; ^10^ Department of Psychiatry Amsterdam UMC Location Vrije Universiteit Amsterdam Amsterdam The Netherlands; ^11^ Peking‐Tsinghua Centre for Life Sciences and PKU‐IDG/McGovern Institute for Brain Research Peking University Beijing China; ^12^ National Institute on Drug Dependence and Beijing Key Laboratory of Drug Dependence Peking University Beijing China; ^13^ Institute of Education & Child Studies, Section Forensic Family & Youth Care Leiden University Leiden The Netherlands

**Keywords:** 7.0 Tesla, functional connectivity, major depressive disorder, structural connectivity, thalamic subnuclei

## Abstract

**Aims:**

Major depressive disorder (MDD) is widely considered to be a mood disorder characterized by altered connectivity. The thalamus plays an important role in MDD by connecting large areas of the brain. Here, we explored thalamic connectivity in MDD at the subnuclear level using ultra‐high‐field MRI.

**Methods:**

We combined ultra‐high‐field functional and diffusion MRI at 7.0 Tesla to map the connectivity of thalamic subnuclei in MDD patients (*n* = 47) and healthy controls (*n* = 13). We segmented thalamic subnuclei and calculated the functional and structural connectivity of thalamic subnuclei and tested for group differences and associations with clinical characteristics.

**Results:**

MDD patients showed increased functional connectivity of the right thalamic central lateral nucleus with the right amygdala, bilateral inferior occipital lobe, and right transverse temporal gyrus, which was accompanied by increased structural connectivity between the right thalamic central lateral nucleus and the right inferior occipital lobe. Medicated MDD patients had a greater streamline count of the right thalamic central lateral nucleus–right inferior occipital lobe tract than healthy controls.

**Conclusions:**

Thalamic subnuclear connectivity with cortical and subcortical brain regions is perturbed in MDD. These results further support increased thalamic connectivity in MDD and suggest that this is related to a specific subnucleus.

The pathological mechanisms of major depressive disorder (MDD) have always been a focal point of research. Despite numerous studies in this area, our understanding of the pathophysiology of MDD remains incomplete.^1^, ^2^ One significant reason for this is the limited spatial precision of current brain research technologies. Magnetic resonance imaging (MRI) is an essential tool for studying the living brain. However, most previous MRI studies exploring the neural imaging mechanisms in MDD patients have utilized conventional magnetic fields (1.5/3.0 Tesla), leading to the oversight of many details.^3^ The high signal‐to‐noise ratio of 7.0 Tesla MRI allows for a clearer exploration of emotional pathways.^4^ Here, we employed ultra‐high‐field (UHF) MRI to investigate the brain characteristics of MDD patients.

Among the various neuropathological mechanisms of MDD, the thalamus holds a crucial position.^5^, ^6^, ^7^, ^8^ Increasing evidence suggests that MDD should be understood as a disorder of imbalanced brain connectivity.^9^, ^10^ Given that the thalamus serves as a central relay with widespread connections to nearly all cortical and subcortical structures,^11^, ^12^ thalamic connectivity warrants particular attention in MDD research. A recent multi‐site study using deep learning techniques pinpointed thalamic functional hyperconnectivity as the most significant neurophysiological feature of MDD.^13^ Moreover, functional connectivity (FC) between the thalamus and the primary somatosensory cortex is increased in MDD patients.^14^ A positive correlation was found between thalamic‐temporal cortex FC and symptom severity.^15^ In terms of structural connectivity (SC), abnormalities in thalamic white matter connections also appear to varying degrees.^16^, ^17^ Collectively, these findings motivate further investigation into the role of thalamic connectivity in MDD. In addition, the thalamus consists of numerous subnuclei that connect to different brain regions, each performing distinct functions.^18^ However, most of the previous MRI studies exploring thalamic connectivity in MDD have been conducted at standard field strengths (1.5/3.0 Tesla), overlooking the intricate connections of thalamic subnuclei. Functional studies at conventional field strengths used whole‐thalamus seeds to assess thalamic connectivity.^14^, ^15^ Diffusion studies examined thalamic pathways without separating subnuclei.^16^, ^17^ These approaches lack the spatial resolution required to study subnuclear organization. UHF MRI at 7.0 Tesla provides a markedly higher signal‐to‐noise ratio and spatial precision than 1.5/3.0 Tesla, improving visualization of small thalamic nuclei and their microstructure,^3^, ^4^, ^19^ which allows reliable segmentation of thalamic subnuclei.^18^, ^20^, ^21^ Moreover, 7.0 Tesla functional MRI can reveal MDD‐related disturbances in emotion processing circuitry that standard 3.0 Tesla MRI fails to detect.^4^ Similarly, relative to 3.0 Tesla, the increased diffusion sensitivity at UHF provides a stronger signal and more reliable diffusion tensor imaging measurements, enabling a better fit to the tensor model and more precise tensor estimation.^19^ In sum, compared with traditional field strengths, UHF MRI not only improves the precision of delineating small thalamic subnuclei but also enhances the sensitivity of functional imaging and the accuracy of diffusion imaging.^4^, ^19^, ^20^, ^21^ Therefore, the power of UHF MRI seems warranted to robustly explore the role of the thalamus and its subnuclear connectivity in MDD.

In this study, we therefore employed UHF functional MRI to explore the FC of thalamic subnuclei in MDD patients, for the very first time. Considering the scarcity of studies combining multimodal approaches to explore the pathophysiology of MDD,^22^, ^23^ we further employed UHF diffusion MRI to investigate whether these FC abnormalities are supported by SC alterations. Furthermore, the relationships of these FCs and SCs with clinical characteristics were explored.

## Methods

### Participants

The implementation of this study follows the approach used in our previous work on the same dataset,^24^, ^25^, ^26^ while the functional MRI data have not been reported previously. Patients with MDD recruited in this study met the following inclusion criteria: (i) a primary diagnosis of current MDD that occurred within the last 6 months, according to DSM‐5 criteria, as determined by the Composite International Diagnostic Interview (CIDI)^27^; (ii) aged between 20 and 55. Healthy controls (HCs) met the following inclusion criteria: (i) no history of depression diagnosis or treatment, nor any other psychopathology; (ii) normal or subclinical scores on dimensional measures of psychopathology; (iii) aged between 20 and 55. Exclusion criteria for the entire sample included: (i) presence of psychoses, mania, Tourette's syndrome, or obsessive‐compulsive disorder (except anxiety disorder); (ii) diagnosis of major internal or neurological disorders; (iii) traumatic head injury; (iv) current substance abuse or dependence requiring treatment; (v) evidence of acute suicidal risk requiring immediate intervention; (vi) MRI contraindications, including metal implants, heart arrhythmia, or claustrophobia; (vii) left‐handedness; (viii) pregnancy; (ix) inadequate understanding of the Dutch language.

Seventy‐one participants were enrolled in our study (57 patients with MDD and 14 HCs). In the MDD group, data from two patients were excluded due to missing fMRI data, data from one patient were excluded following quality control of the T1 image, and data from seven patients were excluded following quality control of the fMRI data. In the HC group, data from one participant were excluded after quality control of the fMRI data. The final sample consisted of 47 participants with a primary diagnosis of MDD (mean age = 36.79 years, SD = 10.67 years, 35 females) and 13 HC participants (mean age = 35.97 years, SD = 9.34 years, 8 females). Detailed demographic and clinical information is presented in Table 1.

**Table 1:** Demographical and clinical information of the participants^†^

	MDD (*n* = 47)	HC (*n* = 13)	*t/χ* ^2^	*P* ^‡^
Age (years), mean ± SD	36.79 ± 10.67	35.97 ± 9.34	−0.27	0.788
Gender (female), *n* (%)	35 (74.47%)	8 (61.54%)	0.84	0.360
Age of onset (years), mean ± SD	21.15 ± 10.50	—		—
Recurrent depression, *n* (%)	20 (42.55%)	—		—
Comorbid anxiety disorder, *n* (%)	20 (42.55%)	—		—
IDS^§^, mean ± SD	33.96 ± 13.34	4.08 ± 2.93	−14.18	**<0.001**
BAI^§^, mean ± SD	14.13 ± 9.82	2.15 ± 2.38	−7.60	**<0.001**
IRS^§^, mean ± SD	10.04 ± 5.08	5.62 ± 4.29	−3.16	**0.005**
CTQ^§^, mean ± SD	48.19 ± 18.23	38.00 ± 9.13	−2.78	**0.008**
With any psychotropic medications, *n* (%)	26 (55.32%)	—		—
With any antidepressants, *n* (%)	23 (48.94%)	—		—
With SSRIs and/or SNRIs, *n* (%)	18 (38.30%)	—		—
With TCA, *n* (%)	6 (12.77%)	—		—
With atypical antidepressants, *n* (%)	2 (4.26%)	—		—
With lithium stabilizer, *n* (%)	3 (6.38%)	—		—
With antipsychotics, *n* (%)	5 (10.64%)	—		—
With benzodiazepines, *n* (%)	5 (10.64%)	—		—

^†^ Group differences were tested with Welch's *t*‐test or chi‐square test.

^‡^ Significance: *P* < 0.05. Bold indicates significant *P*‐value.

^§^ One MDD patient did not provide the data for IDS, BAI, IRS, and CTQ. Therefore, when analyzing the data related to these indicators, the sample size for MDD is 46 individuals.

BAI, Beck Anxiety Inventory; CTQ, Childhood Trauma Questionnaire; HC, healthy control; IDS, Inventory for Depressive Symptomatology; IRS, Insomnia Rating Scale; MDD, major depressive disorder; SD, standard deviation; SNRI, serotonin‐norepinephrine reuptake inhibitor; SSRI, selective serotonin reuptake inhibitor; TCA, tricyclic antidepressant.

For the dMRI part, detailed demographic and clinical information are presented in Table S1. The medical ethical review board of the Amsterdam UMC (location VUmc) approved this study, and written informed consent was obtained from all participants.

### Clinical measures

The Inventory for Depressive Symptomatology (IDS),^28^ the Beck Anxiety Inventory (BAI),^29^ the Insomnia Rating Scale (IRS),^30^ and the Childhood Trauma Questionnaire (CTQ)^31^ were used to measure the severity of depression, anxiety, insomnia, and childhood trauma, respectively.

### MRI acquisition

Images were acquired using a Philips Achieva 7.0 Tesla MRI scanner with a 32‐channel head array coil. Resting‐state fMRI data were collected for intrinsic FC estimates using the following parameters: echo time (TE) = 18.6 ms, repetition time (TR) = 2000 ms, field of view (FOV) = 192 × 92.25 × 192 mm, flip angle = 60°, spatial resolution = 1.5 × 1.5 × 1.65 mm. During the resting‐state fMRI scan, participants were instructed to close their eyes without falling asleep. Furthermore, participants were asked whether they stayed awake after finishing the scan. The total acquisition time for the resting‐state fMRI data was 10 min (300 volumes).

Diffusion‐weighted imaging (DWI) was additionally implemented for SC estimates using the following parameters: TE = 74 ms, TR = 11,224 ms, FOV = 214 × 126.5 × 214 mm, flip angle = 90°, spatial resolution = 1.337 × 1.337 × 1.49 mm, and the number of gradient directions = 64. The b‐value for the sequence was 1200 s/mm^2^, and one b = 0 s/mm^2^ was acquired, with a total acquisition time = 13.57 min. Additionally, two non‐diffusion‐weighted (b = 0) images with opposite phase‐encoding directions were acquired to correct for gradient distortions.^32^

Finally, T1‐weighted images were obtained for anatomical referencing using an MP2RAGEME (multi‐echo magnetization‐prepared rapid gradient echo) sequence.^33^ The MP2RAGEME is an extension of the MP2RAGE sequence^34^ and consists of two rapid gradient echo (GRE_1,2_) images that are acquired after a 180° inversion pulse and excitation pulses with inversion times TI_1,2_ = [670 ms, 3675.4 ms]. A multi‐echo readout was used in the second inversion, with four equally spaced echo times (TE_1_ = 3 ms, TE_2,1–4_ = 3, 11.5, 19, 28.5 ms). Other scan parameters include flip angle_1,2_ = [4°,4°]; TR_GRE1,2_ = [6.2 ms, 31 ms]; bandwidth = 404.9 MHz; TR_MP2RAGEME_ = 6778 ms; acceleration factor SENSE_PA_ = 2; FOV = 205 × 205 × 164 mm; acquired voxel size = 0.7 × 0.7 × 0.7 mm; acquisition matrix was 292 × 290; reconstructed voxel size 0.64 × 0.64 × 0.7 mm; turbo factor (TFE) = 150, resulting in 176 shots; total acquisition time = 19.53 min.

### Atlas construction

The Montreal Neurological Institute 152‐subject average T1‐weighted MRI template with 0.5 mm isotropic resolution (https://www.mcgill.ca/bic/resources/brain-imaging-citrix) and the individual T1‐weighted images were segmented to create the atlas for fMRI and dMRI data extraction. The T1‐weighted images were preprocessed using the “recon‐all” pipeline with FreeSurfer version 7.4.0 (http://surfer.nmr.mgh.harvard.edu). The preprocessing steps include normalization of signal intensity, skull‐stripping, Talairach transformation, and automated segmentation of subcortical white matter and gray matter structures.^35^

**Fig. 1:**
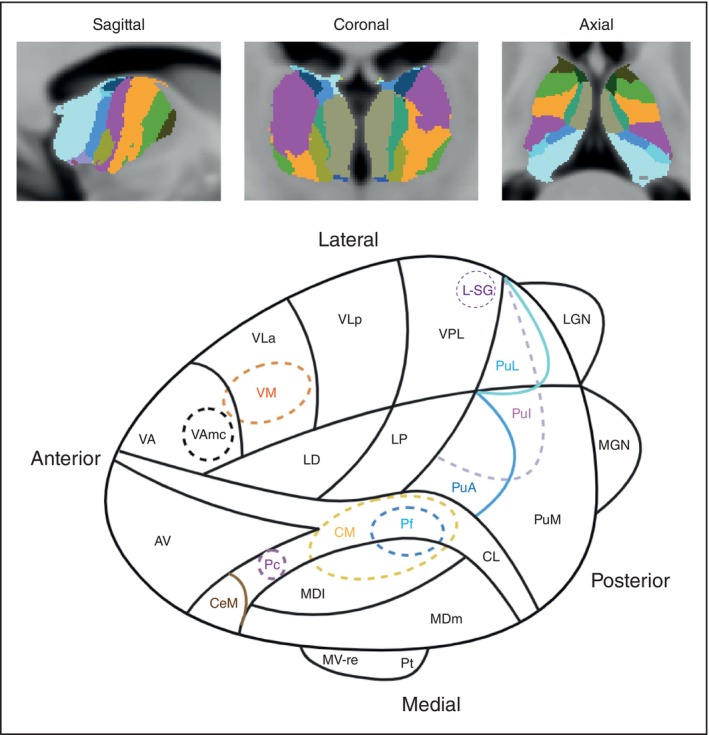
Subfields of the thalamus. AV, anteroventral nucleus; CeM, central medial nucleus; CL, central lateral nucleus; CM, centromedian nucleus; L‐SG, limitans‐suprageniculate nucleus; LD, laterodorsal nucleus; LGN, lateral geniculate nucleus; LP, lateral posterior nucleus; MDI, mediodorsal lateral parvocellular nucleus; MDm, mediodorsal medial magnocellular nucleus; MGN, medial geniculate nucleus; MV‐re, medial ventral reuniens nucleus; Pc, paracentral nucleus; Pf, parafascicular nucleus; PuA, pulvinar anterior nucleus; PuI, pulvinar inferior nucleus; PuL, pulvinar lateral nucleus; PuM, pulvinar medial nucleus; Pt, paratenial nucleus; VA, ventral anterior nucleus; VAmc, ventral anterior magnocellular nucleus; Vla, ventral lateral anterior nucleus; VLp, ventral lateral posterior nucleus; VM, ventromedial nucleus; VPL, ventral posterolateral nucleus. Reprinted with permission from Springer Publishing: European Archives of Psychiatry and Clinical Neuroscience (W. Liu *et al*., 2025).

Afterward, the preprocessed T1‐weighted images were subjected to thalamic segmentation using the subfield segmentation module of FreeSurfer, which demonstrated excellent test–retest reliability and remained robust to variations in input MRI contrast.^18^, ^36^, ^37^ In this step, as shown in Fig. 1, the thalamus was segmented into 25 different subfields: anteroventral, laterodorsal, lateral posterior, ventral anterior, ventral anterior magnocellular, ventral lateral anterior, ventral lateral posterior, ventral posterolateral, ventromedial, centromedian, central medial, central lateral, paracentral, parafascicular, paratenial, medial ventral reuniens, mediodorsal medial magnocellular, mediodorsal lateral parvocellular, lateral geniculate, medial geniculate, limitans‐suprageniculate, pulvinar anterior, pulvinar medial, pulvinar lateral, and pulvinar inferior nuclei. Since not all participants' bilateral ventromedial, paracentral, and paratenial nuclei could be identified in the segmentation, these three nuclei were excluded. Segmentations were inspected manually.

Since we found a significant group effect (MDD/HC) in FC between the right central lateral nucleus and the entire right amygdala, we further analyzed which specific amygdala subregions were responsible for this significant group effect. For this analysis, the amygdala was segmented into nine subregions: lateral, basal, accessory basal, central, medial, cortical and paralaminar nucleus, and cortico‐amygdaloid transition area and anterior amygdala areas (Fig. S1).

### Resting‐state data preprocessing

Resting‐state fMRI data were processed with fMRIPrep version 20.1.1, which is based on Nipype 1.5.0.^38^, ^39^ Processing of the T1‐weighted anatomical data that served as the reference template for the functional data involved non‐uniformity intensity correction, skull‐stripping, segmentation into white matter, gray matter, and cerebrospinal fluid, and normalization to MNI space at 0.7 mm isotropic resolution. Functional data preprocessing involved skull stripping, six‐parameter head‐motion estimation, slice‐time correction, and susceptibility distortion correction. Co‐registration of functional to anatomical images was subsequently conducted using boundary‐based registration and the six head‐motion parameters, followed by resampling into standard space at 1.5 mm resolution. Various signal confounds were estimated, including Power and Jenkinson framewise displacement, DVARS, and global tissue signals. Participants were defined as motion outliers and excluded from further analysis if more than 20% of the total volumes had a framewise displacement of ≥0.5 mm and/or 1.5 standardized DVARS. Component‐based physiological and temporal regressors were extracted by CompCor following temporal high‐pass filtering at 0.01 Hz. All confounds were subsequently removed from the preprocessed BOLD time courses using pybest,^40^ which first decomposed the extracted noise regressors into 10 principal components. Voxel‐wise removal of these principal components from the time courses was done using fivefold cross‐validation to estimate the optimal number of principal components to remove. Details of resting‐state data preprocessing are presented in Supporting information Methods.

### Diffusion data preprocessing

In line with our previous UHF dMRI study,^24^ diffusion data were processed with MRtrix version 3.0.4 and FSL version 6.0.5.2. The diffusion images were subjected to denoising and Gibbs ringing removal using MRtrix.^41^, ^42^, ^43^, ^44^ FSL was employed for correcting susceptibility‐induced distortions, eddy currents, and motion artifacts.^32^, ^45^, ^46^ Additionally, inhomogeneity field estimation was performed by ANTS N4, and inhomogeneity correction was applied to the DWI volume series.^47^ Afterward, the corrected diffusion‐weighted images were co‐registered to T1‐weighted space using FLIRT, a tool available in FSL.^48^, ^49^ MRtrix was utilized to generate a segmented tissue image, along with a mask image suitable for seeding streamlines at the gray matter–white matter interface.^50^ The estimation of the diffusion (kurtosis) tensor was performed based on weighted least‐squares,^51^, ^52^ while the response function was estimated for spherical deconvolution.^53^ We employed spherical deconvolution to estimate fiber orientation distributions (FOD) from the diffusion data.^54^ For microstructure analysis of DTI measures, we locally fitted the diffusion tensor in each voxel, resulting in whole‐brain maps encompassing FA (a diffusion coherence index), AD (diffusion magnitude in the primary direction), MD (the total diffusion magnitude averaged across measured directions), and RD (the averaged diffusion magnitude in orthogonal secondary and tertiary directions).^55^

### FC calculation

The FC analysis of preprocessed functional images was conducted using the MATLAB‐based pipeline toolbox BRANT (https://github.com/kbxu/brant).^56^ Initially, the average time series of thalamic subnuclei was extracted along with the average time series of the rest of the brain regions of interest (ROIs) in the constructed atlas. By computing the Pearson correlation coefficient between the average time series of the ROIs and applying Fisher's *r* to *z* transformation, we obtained the FC diagram for each individual. In the initial step of our analysis, we discovered a significant group effect (MDD/HC) in the FC between the right central lateral nucleus and the entire right amygdala. To further understand which amygdala subregions were responsible for this result, the same method was subsequently applied to extract the FC values of the right central lateral nucleus and nine right amygdala subregions.

### Tractography

Unlike our previous study that used thalamic subnuclei as seed points for whole‐brain tracking,^25^ we performed point‐to‐point fiber tracking between corresponding brain regions to examine whether abnormalities in FC coincide with changes in SC. Probabilistic tractography was carried out using the iFOD2 algorithm in MRtrix,^50^, ^57^ and thalamic seed regions were selected based on FC group effects, with 500,000 random seeds initiated. The ipsilateral hemisphere mask was employed to restrict the streamline generation. To control streamline generation, we set a cutoff for FOD magnitude at 0.01, limited the maximum angle between consecutive streamline generation steps to 60°, and set the step size as 0.05 according to previous studies.^58^, ^59^, ^60^, ^61^ From each voxel along every streamline, microstructural measurements including FA, MD, RD, and AD were extracted and averaged to obtain a single mean value per participant within each thalamic subnucleus ROI.^62^ To ensure greater accuracy in representing the white matter's ground truth and to eliminate extraneous streamlines, the process of extracting streamline count incorporates spherical deconvolution (SIFT2) weights.^63^ Streamline count was computed as the sum of SIFT2 streamline weights.

In the current study, the right thalamic central lateral nucleus‐left inferior occipital lobe SC was not reconstructed due to the lack of a reasonable anatomical connection.

### Statistical analysis

The scipy package in Python was used to perform the statistical analyses. For demographic and clinical data comparisons, the chi‐square test was used for dichotomous variables and the Welch's *t*‐test for continuous variables. Analysis of covariance with Welch's *t*‐test was performed to investigate the differences in FC and corresponding SC (FA, MD, RD, AD, and streamline count) between groups (MDD/HC). We used Welch's *t*‐test because it provides robust Type I error control under unequal sample sizes and potential variance heterogeneity.^64^, ^65^

Considering that tractography‐derived SC can be influenced by tractography parameters as well as geometric factors such as ROI size and inter‐regional distance,^66^, ^67^, ^68^, ^69^ two additional sensitivity analyses were conducted focusing on the only connection showing a significant group difference in SC. (i) Robustness to tractography parameters: the entire point‐to‐point tractography and SC extraction were repeated while varying key tracking parameters within a plausible range, including the FOD amplitude cutoff (0.015 and 0.005), seeding density (400,000 and 600,000), step size (0.4 and 0.6), and maximum curvature (45° and 75°). We changed one parameter at a time, based on the original parameters, and then re‐analyzed, for a total of eight changes. (ii) Robustness to ROI volume and inter‐regional distance/length: to test robustness to ROI size and length‐related biases, SC was recomputed using MRtrix connectome scaling to account for ROI volume (‐scale_invnodevol) and streamline length effects (‐scale_length).^70^

For those FC and SC showing significant group effect (MDD/HC), partial correlation analysis was conducted to investigate the relationship between FC and SC. Furthermore, partial correlation analyses examined whether the severity of depression, anxiety symptoms, insomnia, and childhood trauma was associated with FC and SC metrics among MDD patients. We also performed the subgroup analysis using Welch's *t*‐test to investigate if the FC and SC differed between medicated MDD, non‐medicated MDD participants, and HCs. Additionally, Welch's *t*‐test was performed to investigate the differences in demographical and clinical characteristics between medicated and non‐medicated MDD patients to show more information.

The above FC and SC analyses were corrected for age, gender, and intracranial volume (ICV). For the FC analyses, the mean framewise displacement was included as an additional covariate in all group‐level and FC‐clinical association analyses. We applied a square root transformation to normalize the highly skewed clinical data. False discovery rate (FDR) correction was implemented to control type I error with a significance level set at alpha = 0.05, and was applied separately within each pre‐specified family of tests. For the group difference analysis of whole‐brain FC, FDR correction was applied across 8,184 tests (44 thalamic subnuclei × 186 ROIs in the constructed atlas). For the follow‐up group difference analysis of right central lateral nucleus‐right amygdala subregions FC, FDR correction was applied across the nine tests (the right central lateral nucleus × nine right amygdala subregions). For the group difference analysis of SC, FDR correction was applied across 25 comparisons (five reconstructed tracts × five SC metrics [FA, MD, RD, AD, and streamline count]). For the sensitivity analyses of the tractography parameters, FDR correction was applied across eight comparisons (eight sets of tractography parameters). For brain‐behavior analyses in the MDD group, FDR correction was applied across 28 partial correlations (seven connectivity metrics [six FC and one SC metrics] × four clinical measures [IDS, BAI, IRS, and CTQ]). Finally, medication subgroup analyses were corrected across 21 tests (three groups [medicated MDD, non‐medicated MDD, and HC] × seven connectivity metrics [six FC and one SC metrics]).

## Results

### Demographical and clinical features

As shown in Table 1, the MDD group comprised 35 females (74.47%), while the HC group included 8 females (61.54%). Among the MDD patients, 20 individuals had recurrent depression (42.55%), with a mean age of onset of 21.15 ± 10.50 years. The Welch's *t*‐test and the chi‐square test showed that there were no group differences in age and gender (*P*s > 0.05). MDD patients had significantly higher scores on the IDS, BAI, IRS, and CTQ (*P*s < 0.05). Demographical and clinical details of the dMRI are shown in Table S1.

### MDD diagnosis and clinical characteristics related to thalamic subnuclei connectivity

**Fig. 2:**
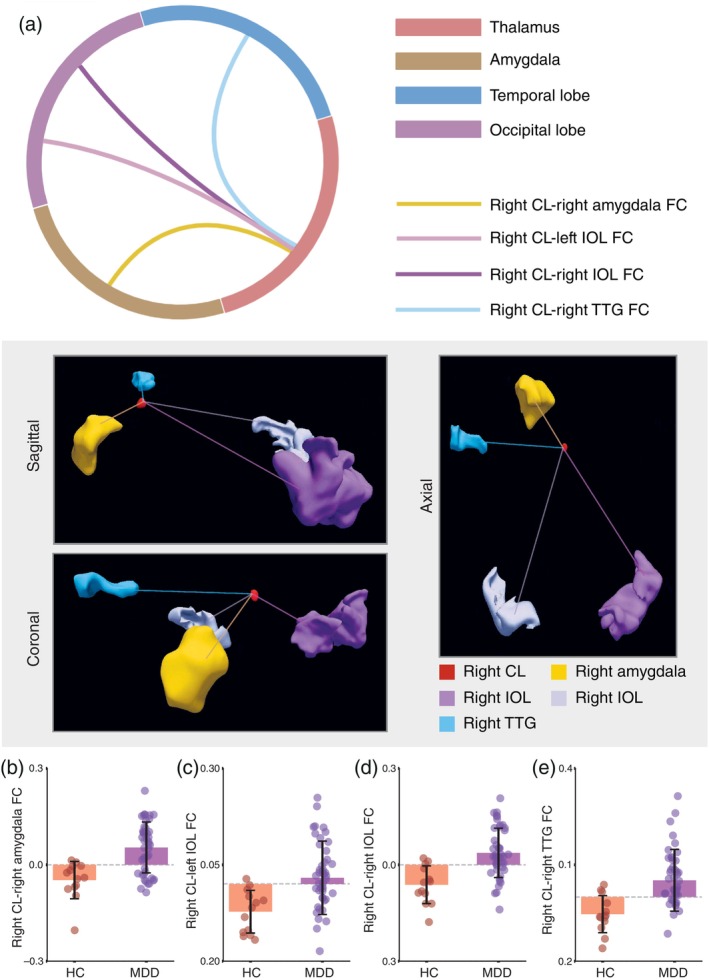
Significant differences in FCs of the central lateral nucleus between MDD and HC. (a) FCs of the central lateral nucleus with significant differences between MDD and HC. Orange is the right CL‐right amygdala FC, light purple is the right CL‐left IOL FC, dark purple is the right CL‐right IOL FC, and blue is the right CL‐right TTG FC. (b) MDD participants had higher right CL‐right amygdala FC than HCs (*t* = −5.45, d.f. = 25.01, *P*_FDR_ = 0.038, 95% CI [−0.14, −0.07], Cohen's *d* = −1.57). (c) MDD participants had higher right CL‐left IOL FC than HCs (*t* = −5.05, d.f. = 30.97, *P*_FDR_ = 0.038, 95% CI [−0.14, −0.06], Cohen's *d* = −1.37). (d) MDD participants had higher right CL‐right IOL FC than HCs (*t* = −5.51, d.f. = 25.83, *P*_FDR_ = 0.038, 95% CI [−0.15, −0.07], Cohen's *d* = −1.57). (e) MDD participants had higher right CL‐right TTG FC than HCs (*t* = −4.98, d.f. = 35.11, *P*_FDR_ = 0.038, 95% CI [−0.14, −0.06], Cohen's *d* = −1.31). Group differences were tested using Welch's *t*‐test (two‐sided) with age, gender, ICV, and mean framewise displacement as covariates. FDR correction was applied across 8,184 tests (44 thalamic subnuclei × 186 ROIs in the constructed atlas). Significance: *P*_FDR_ < 0.05. CL, central lateral nucleus; FC, functional connectivity; HC, healthy control; IOL, inferior occipital lobe; MDD, major depressive disorder; TTG, transverse temporal gyrus.

Analysis of FC revealed increased right thalamic central lateral nuclear connectivity with the right amygdala (*t* = −5.45, d.f. = 25.01, *P*_FDR_ = 0.038, 95% CI [−0.14, −0.07], Cohen's *d* = −1.57; Fig. 2a,b) and bilateral inferior occipital lobe in MDD patients relative to HC participants (left: *t* = −5.05, d.f. = 30.97, *P*_FDR_ = 0.038, 95% CI [−0.14, −0.06], Cohen's *d* = −1.37; Fig. 2a,c; right: *t* = −5.51, d.f. = 25.83, *P*_FDR_ = 0.038, 95% CI [−0.15, −0.07], Cohen's *d* = −1.57; Fig. 2a,d). Moreover, MDD patients exhibited increased right thalamic central lateral nuclear connectivity with the right transverse temporal gyrus (*t* = −4.98, d.f. = 35.11, *P*_FDR_ = 0.038, 95% CI [−0.14, −0.06], Cohen's *d* = −1.31; Fig. 2a,e).

**Fig. 3:**
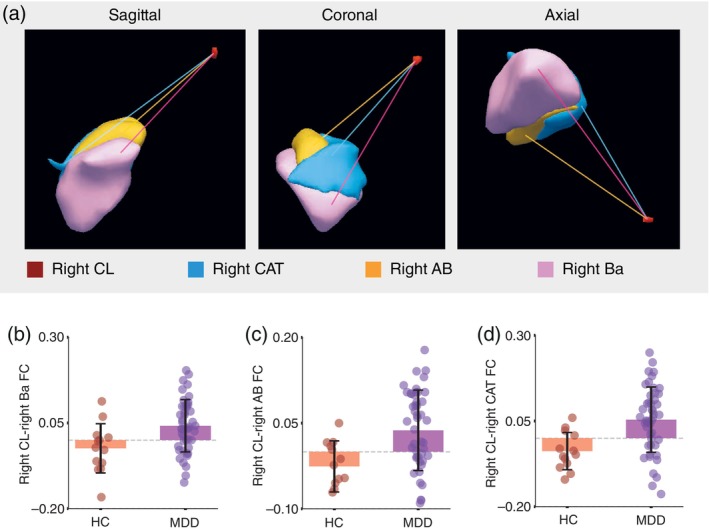
Significant differences in FCs of central lateral nucleus‐amygdala subfields between MDD and HC. (a) FCs of central lateral nucleus‐amygdala subfields with significant differences between MDD and HC. Red is the right CL‐right Ba FC, orange is the right CL‐right AB FC, and blue is the right CL‐right CAT FC. (b) MDD participants had higher right CL‐right Ba FC than HCs (*t* = −4.14, d.f. = 27.20, *P*_FDR_ = 0.001, 95% CI [−0.11, −0.04], Cohen's *d* = −1.16). (c) MDD participants had higher right CL‐right AB FC than HCs (*t* = −3.97, d.f. = 31.90, *P*_FDR_ = 0.001, 95% CI [−0.09, −0.03], Cohen's *d* = −1.07). (d) MDD participants had higher right CL‐right CAT FC than HCs (*t* = −4.37, d.f. = 27.11, *P*_FDR_ = 0.001, 95% CI [−0.14, −0.05], Cohen's *d* = −1.23). Group differences were tested using Welch's *t*‐test (two‐sided) with age, gender, ICV, and mean framewise displacement as covariates. FDR correction was applied across the nine tests (the right central lateral nucleus × nine right amygdala subregions). Significance: *P*_FDR_ < 0.05. AB, accessory basal nucleus; Ba, basal nucleus; CAT, cortico‐amygdaloid transition area; CL, central lateral nucleus; FC, functional connectivity; HC, healthy control; MDD, major depressive disorder.

After segmenting the amygdala, we found that participants with MDD had significantly greater right thalamic central lateral nucleus‐right amygdala basal nucleus FC (*t* = −4.14, d.f. = 27.20, *P*_FDR_ = 0.001, 95% CI [−0.11, −0.04], Cohen's *d* = −1.16; Fig. 3a,b), right thalamic central lateral nucleus‐right amygdala accessory basal nucleus (*t* = −3.97, d.f. = 31.90, *P*_FDR_ = 0.001, 95% CI [−0.09, −0.03], Cohen's *d* = −1.07; Fig. 3a,c), and right thalamic central lateral nucleus‐right cortico‐amygdaloid transition area (*t* = −4.37, d.f. = 27.11, *P*_FDR_ = 0.001, 95% CI [−0.14, −0.05], Cohen's *d* = −1.23; Fig. 3a,d) than HCs.

**Fig. 4:**
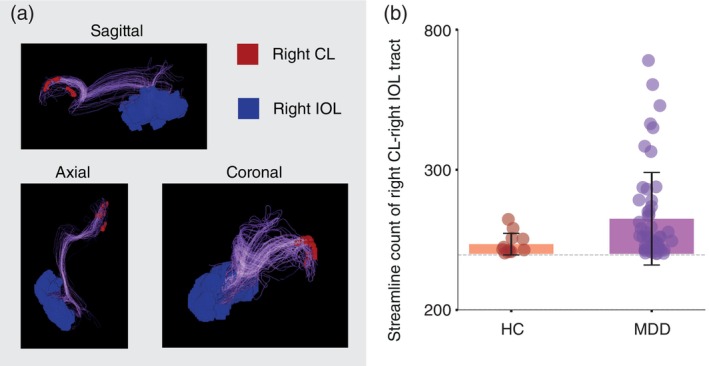
Difference in streamline count in the tract between the right central lateral nucleus and right inferior occipital lobe between the MDD and HC groups. (a) The right CL‐right IOL tract. (b) Bar chart of the streamline count right CL‐right IOL tract between MDD and HC group (*t* = −3.43, d.f. = 63.00, *P*_FDR_ = 0.021, 95% CI [−131.28, −34.63], Cohen's *d* = −0.72). Streamline count was quantified using SIFT2‐weighted. Group differences were tested using Welch's *t*‐test (two‐sided) with age, gender, and ICV as covariates. FDR correction was applied across 25 comparisons (five reconstructed tracts × five SC metrics [FA, MD, RD, AD, and streamline count]). Significance: *P*_FDR_ < 0.05. CL, central lateral nucleus; HC, health control; IOL, inferior occipital lobe; MDD, major depressive disorder.

Because of the central role of the right thalamic central lateral nucleus in increased FC, we additionally investigated the corresponding SC. Group comparisons showed significantly higher white matter fiber streamline count of the right thalamic central lateral nucleus‐right inferior occipital lobe SC in MDD patients (*t* = −3.43, d.f. = 63.00, *P*_FDR_ = 0.021, 95% CI [−131.28, −34.63], Cohen's *d* = −0.72; Table 2, Fig. 4), thus pointing to coinciding increased FC and SC within this circuit. Other SCs with no significant difference are shown in Fig. S2.

**Table 2:** White matter features in tracts spanning from the central lateral nucleus in the right hemisphere of MDD patients and HCs^†^ ^,^ ^‡^

Tracts	MDD (*n* = 53)	HC (*n* = 12)	*t*	d.f.	*P*_FDR_ ^§^	95% CI	Cohen's *d*
FA of CL‐IOL tract	0.43 ± 0.02	0.43 ± 0.04	−0.39	26.01	0.870	[−0.02, 0.01]	−0.11
FA of CL‐Ba tract	0.38 ± 0.03	0.37 ± 0.04	1.13	22.42	0.512	[−0.01, 0.03]	0.32
FA of CL‐AB tract	0.34 ± 0.04	0.34 ± 0.03	0.08	14.56	0.935	[−0.02, 0.03]	0.03
FA of CL‐CAT tract	0.36 ± 0.04	0.35 ± 0.04	1.21	16.82	0.512	[−0.01, 0.04]	0.38
MD of CL‐IOL tract	0.43 ± 0.02	0.43 ± 0.04	−0.39	26.01	0.871	[−0.02, 0.01]	−0.11
MD of CL‐Ba tract	0.38 ± 0.03	0.37 ± 0.04	1.13	22.42	0.512	[−0.01, 0.03]	0.32
MD of CL‐AB tract	0.34 ± 0.04	0.34 ± 0.03	0.08	14.56	0.935	[−0.02, 0.03]	0.03
MD of CL‐CAT tract	0.36 ± 0.04	0.35 ± 0.04	1.21	16.82	0.512	[−0.01, 0.04]	0.38
RD of CL‐IOL tract	0.43 ± 0.02	0.43 ± 0.04	−0.39	26.01	0.871	[−0.02, 0.01]	−0.11
RD of CL‐Ba tract	0.38 ± 0.03	0.37 ± 0.04	1.13	22.42	0.512	[−0.01, 0.03]	0.32
RD of CL‐AB tract	0.34 ± 0.04	0.34 ± 0.03	0.08	14.56	0.935	[−0.02, 0.03]	0.03
RD of CL‐CAT tract	0.36 ± 0.04	0.35 ± 0.04	1.12	16.82	0.512	[−0.01, 0.04]	0.38
AD of CL‐IOL tract	0.43 ± 0.02	0.43 ± 0.04	−0.39	26.01	0.871	[−0.02, 0.01]	−0.11
AD of CL‐Ba tract	0.38 ± 0.03	0.37 ± 0.04	1.13	22.42	0.512	[−0.01, 0.03]	0.32
AD of CL‐AB tract	0.34 ± 0.04	0.34 ± 0.03	0.08	14.56	0.935	[−0.02, 0.03]	0.03
AD of CL‐CAT tract	0.36 ± 0.04	0.35 ± 0.04	1.21	16.82	0.512	[−0.01, 0.04]	0.38
SLC of CL‐IOL tract	34.75 ± 38.46	125.32 ± 165.16	−3.43	63.00	**0.021**	[−131.28, −34.63]	−0.72
SLC of CL‐Ba tract	40.75 ± 55.73	97.25 ± 164.35	−1.10	24.98	0.512	[−103.40, 31.40]	−0.30
SLC of CL‐AB tract	27.33 ± 33.23	49.34 ± 86.56	−0.73	47.71	0.778	[−40.12, 18.68]	−0.17
SLC of CL‐CAT tract	587.75 ± 1026.04	1784.45 ± 3991.38	−1.47	58.57	0.512	[−2139.36, 325.07]	−0.32

^†^ Group differences were tested using Welch's *t*‐test (two‐sided) with age, gender, and ICV as covariates.

^‡^ FDR correction was applied across 25 comparisons (five reconstructed tracts × five SC metrics [FA, MD, RD, AD, and streamline count]).

^§^ Significance: *P*_FDR_ < 0.05. Bold indicates significant *P*_FDR_ value.

AB, accessory basal nucleus; AD, axial diffusivity; Ba, basal nucleus; CAT, cortico‐amygdaloid transition area; CL, central lateral nucleus; FA, fractional anisotropy; HC, healthy control; IOL, inferior occipital lobe; MD, mean diffusivity; MDD, major depressive disorder; RD, radial diffusivity; SLC = streamline count.

For the sensitivity analyses, (i) after changing the tractography parameters, group effect in streamline count of the right thalamic central lateral nucleus‐right inferior occipital lobe SC persisted under these alternatives (*P*_FDR_s < 0.01; Table S2); (ii) while controlling the ROI volume and inter‐regional distance/length, the group effect remained (*t* = −3.73, d.f. = 62.87, *P* < 0.001, 95% CI [−0.21, −0.06], Cohen's *d* = −0.61).

However, there was no significant association between FC and streamline count of the right thalamic central lateral nucleus‐right inferior occipital lobe connectivity (*r* = −0.12, *P* = 0.46, 95% CI [−0.42, 0.16], Fisher's *z* = −0.12).

We subsequently investigated the differences in these FC and SC measures between medicated and non‐medicated MDD, and found no significant differences (*P*_FDR_s > 0.05). Both medicated and non‐medicated MDD had higher FC than HC (*P*_FDR_s < 0.01). However, a significantly greater streamline count of the right thalamic central lateral nucleus‐right inferior occipital lobe tract was found in medicated MDD (*t* = 3.13, d.f. = 33.97, *P*_FDR_ = 0.007, 95% CI [37.89, 178.53], Cohen's *d* = 0.83) but not in non‐medicated MDD (*t* = 1.82, d.f. = 28.15, *P*_FDR_ = 0.111, 95% CI [−6.28, 106.31], Cohen's *d* = 0.56) when compared with HC. Details of the results of medicated subgroup analyses are shown in Table 3. Medicated MDD patients had a higher risk of comorbid anxiety disorder, higher IDS scores, and BAI scores compared with non‐medicated patients (*P*s < 0.05, Table S3). Furthermore, none of the associations between FC and SC and clinical characteristics were significant (*P*_FDR_s > 0.05).

**Table 3:** Differences in FC^†^ and SC^‡^ between medicated and non‐medicated MDD^§^ ^,^ ^¶^

	Connectivity	*t*	d.f.	*P*_FDR_ ^††^	95% CI	Cohen's *d*
Medicated versus non‐medicated MDD	Right CL‐right Ba FC	1.20	38.70	0.294	[−0.02, 0.07]	0.36
	Right CL‐right AB FC	0.18	36.73	0.860	[−0.04, 0.05]	0.05
	Right CL‐right CAT FC	0.21	33.41	0.860	[−0.05, 0.06]	0.06
	Right CL‐left IOL FC	−0.22	43.58	0.860	[−0.06, 0.05]	−0.06
	Right CL‐right IOL FC	0.41	44.57	0.794	[−0.04, 0.05]	0.12
	Right CL‐right TTG FC	2.06	37.92	0.070	[0.001, 0.11]	0.61
	SLC of right CL‐right IOL tract	−1.39	50.31	0.223	[−142.08, 25.69]	−0.38
Non‐medicated MDD versus HC	Right CL‐right Ba FC	3.90	31.94	**0.001**	[0.04, 0.13]	1.31
	Right CL‐right AB FC	3.02	31.45	**0.008**	[0.02, 0.10]	0.99
	Right CL‐right CAT FC	3.34	31.90	**0.005**	[0.04, 0.15]	1.11
	Right CL‐left IOL FC	4.01	31.97	**0.001**	[0.05, 0.14]	1.34
	Right CL‐right IOL FC	5.00	30.21	**<0.001**	[0.07, 0.16]	1.71
	Right CL‐right TTG FC	4.96	31.40	**<0.001**	[0.08, 0.19]	1.63
	SLC of right CL‐right IOL tract	1.82	28.15	0.111	[−6.28, 106.31]	0.56
Medicated MDD versus HC	Right CL‐right Ba FC	3.21	30.35	**0.006**	[0.02, 0.10]	1.04
	Right CL‐right AB FC	3.57	32.89	**0.003**	[0.03, 0.09]	1.14
	Right CL‐right CAT FC	4.11	27.47	**0.001**	[0.05, 0.13]	1.36
	Right CL‐left IOL FC	4.41	35.41	**<0.001**	[0.05, 0.15]	1.38
	Right CL‐right IOL FC	4.55	33.06	**<0.001**	[0.06, 0.15]	1.45
	Right CL‐right TTG FC	3.49	34.02	**0.003**	[0.03, 0.12]	1.11
	SLC of right CL‐right IOL tract	3.13	33.97	**0.007**	[37.89, 178.53]	0.83

^†^ Sample size of FC analysis: 26 medicated MDD, 21 non‐medicated MDD, and 13 HC.

^‡^ Sample size of SC analysis: 30 medicated MDD, 23 non‐medicated MDD, and 12 HC.

^§^ Group differences were tested using Welch's *t*‐test (two‐sided) with age, gender, and ICV as covariates for the SC analysis, and age, gender, ICV, and mean framewise displacement as covariates for the FC analysis.

^¶^ FDR correction was applied across 21 tests (three groups [medicated MDD, non‐medicated MDD, and HC] × seven connectivity metrics [six FC and one SC metrics]).

^††^ Significance: *P*_FDR_ < 0.05. Bold indicates significant *P*_FDR_ value.

AB, accessory basal nucleus; Ba, basal nucleus; CAT, cortico‐amygdaloid transition area; CL, central lateral nucleus; FC, functional connectivity; HC, healthy control; IOL, inferior occipital lobe; MDD, major depressive disorder; SC, structural connectivity; SLC, streamline count; TTG, transverse temporal gyrus.

## Discussion

Most of the previous studies on thalamic connectivity used standard field strength MRI (1.5/3.0 Tesla) and considered the thalamus as a whole, overlooking the different functions of its subnuclei. By using the high resolution of UHF MRI, we identified that increased thalamic connectivity in MDD is specifically related to the right thalamic central lateral nucleus, which also showed increased SC. These results provide a deeper understanding of thalamic subnuclear connectivity in MDD.

### Thalamic central lateral nucleus prime effect site of connectivity changes

Prior work points to thalamic hyperconnectivity as a prominent neurophysiological feature of MDD.^13^, ^14^ In our study, thalamic central lateral nucleus connectivity profiles stood out among numerous thalamic subnuclei, both at the functional and SC level. The function of the thalamic central lateral nucleus is primarily related to cognitive processing and arousal,^71^ both of which have been found to be impaired in MDD patients.^1^, ^72^ Cognitive impairment is common in MDD,^1^ often persisting even after remission,^73^, ^74^ and it can also predict the treatment outcome of depression.^75^ Moreover, sleep and arousal disruptions are frequently observed in people experiencing depression.^76^ Those who respond positively to antidepressants tend to exhibit heightened brain wakefulness, indicating that dysregulation of brain arousal could potentially serve as a predictive indicator for treatment response in depression.^72^ By leveraging the power of UHF MRI, our study points to changes in FC and SC of the thalamic central lateral nucleus as a potential marker of MDD, which deserves further verification in subsequent studies.

### Increased thalamic central lateral nucleus connectivity with the amygdala in MDD

In addition to the central lateral nucleus of the thalamus, the amygdala also plays an important role in affective/emotional states and emotional processing.^77^ Our analyses showed that MDD patients have stronger thalamic central lateral nuclear FC with the amygdala than HCs. Furthermore, we found three amygdala subnuclei that stand out in our results: the basal nucleus, accessory basal nucleus, and cortico‐amygdaloid transition area. In previous studies, the amygdala is commonly divided into larger subregions, with the basal nucleus typically included within the basolateral amygdala.^78^, ^79^ Therefore, most studies focus on the basolateral nucleus. Animal studies indicate a close association between basolateral amygdala and chronic stress, a known trigger of depression.^80^, ^81^ A postmortem investigation revealed a higher total count of basolateral amygdala neurovascular cells in MDD individuals compared with HCs.^82^ Additionally, a large‐scale study revealed reduced thickness and surface area of the basolateral amygdala in patients with juvenile‐onset MDD compared with HCs.^83^ Concerning the basal nucleus, a previous study revealed that drug‐naïve patients with first‐episode depression had larger volumes of the basal nucleus on both sides compared with HCs.^84^ Furthermore, the volume of the left basal nucleus showed a negative association with anxiety in participants with depression.^84^ This study also found that the left accessory basal nucleus volume was larger in the depression group compared with the HC group, and positively correlated with anxiety levels among depressed patients.^84^ Previous studies have not reported the relationship between cortico‐amygdaloid transition area and depression. The ventral border of the cortico‐amygdaloid transition area merges posteriorly into the hippocampal‐amygdala transition area,^36^ which is associated with an elevated risk of incident (chronic) depression.^85^ There is overwhelming evidence that the direct or indirect connections between the thalamus and amygdala are closely related to emotions and depression.^86^, ^87^, ^88^ Our study pinpoints the FC between specific thalamic subnuclei as well as amygdala subregions that play a role in MDD. However, we did not detect a corresponding SC difference for the central lateral nucleus‐amygdala tract, suggesting that this effect reflects predominantly functional and/or indirect thalamic‐amygdala coupling.

### Increased thalamic central lateral nucleus connectivity with visual cortex in MDD

Our study found that thalamic central lateral nucleus FC with the inferior occipital lobe was greater in MDD participants than HCs, which crucially also coincided with increased SC between these regions. The inferior occipital lobe is a component of the visual cortex,^89^ which is linked to depression and the efficacy of antidepressant treatment.^90^ The gray matter volume of the visual cortex in patients with MDD is larger than that in HCs.^91^ Moreover, MDD patients exhibit a higher incidence of asymmetric development of the visual cortex compared with HCs.^92^ In a resting‐state fMRI study, MDD patients demonstrated reduced FC within the visual network relative to HCs, and the decrease in FC was positively correlated with the illness duration of MDD.^93^ Emotion regulation task‐fMRI also shows that activity in the right amygdala and visual cortex of HCs is downregulated during negative emotional states, but not in MDD patients.^94^ In our study, UHF MRI was used to verify the role of the thalamus‐visual cortex pathway in MDD, and demonstrated that this pathway may serve as a potential marker of MDD. We used a more refined thalamic division here to demonstrate the role of the thalamic central lateral nucleus in MDD patients.

In addition, our previous study found that SC of thalamic subnuclei was widely reduced in MDD patients,^25^ while specific SC in MDD was increased in the present study with increased FC. Unlike the point‐to‐point tractography in the current study, we performed whole‐brain tractography on thalamic subnuclei in the previous study,^25^ which may have overlooked some details. Moreover, in our previous study, the integration of the thalamic central lateral nucleus into the intralaminar nucleus for analysis may have contributed to the loss of subtle yet meaningful changes. It is worth noting that our SC results remained significant after multiple sensitivity analyses. Therefore, there is reason to believe that the robustness of our results explains the pathological changes overlooked by previous extensive whole‐brain fiber tracking.

Moreover, we found medicated MDD patients had a greater streamline count of the right thalamic central lateral nucleus‐right inferior occipital lobe tract than HC, while we did not find a similar pattern in non‐medicated MDD patients. As medicated MDD patients had a higher risk of comorbid anxiety disorder and had more severe depressive and anxious symptoms (Table S3), we speculate that the complexity and severity of MDD may lead to an increase in the streamline count of the right thalamic central lateral nucleus‐right inferior occipital lobe tract. Further study with a larger sample size should be performed to verify this result.

### Increased thalamic central lateral nucleus connectivity with auditory cortex in MDD

We also found that thalamic central lateral nucleus connectivity with the transverse temporal gyrus, a subregion of the auditory cortex, was abnormally increased in MDD patients relative to HCs. Patients with MDD exhibit functional and morphological anomalies within the auditory cortex,^95^ which seem to track treatment response and efficacy in a subset of MDD patients.^96^ Previous work has moreover shown that the thalamic‐auditory cortex pathway is more pronounced in MDD patients compared with HCs,^15^ largely consistent with our findings.

### The high road and low road in MDD

Using UHF MRI, we identified increased connectivity in the network that is central in the dual pathway hypothesis for emotion processing.^97^ The “low road” refers to a fast subcortical pathway that delivers rapid but relatively crude representations of sensory information directly to the amygdala *via* the thalamus, allowing for quick, automatic responses to potential threats or danger.^98^ The “high road” is a slower cortical pathway that involves more complex processing through sensory cortices and higher‐order brain regions before reaching the amygdala. This pathway enables more detailed analysis and evaluation of sensory information, leading to more nuanced emotional responses.^99^ In the present study, we found that MDD patients showed increased FC of the right thalamic central lateral nucleus with the right amygdala, bilateral inferior occipital lobe (visual cortex component), and the right transverse temporal gyrus (auditory cortex component). Specifically, the increased right thalamic central lateral nucleus‐right amygdala FC is interpreted as a low‐road‐like thalamic‐amygdala functional coupling, whereas the increased right thalamic central lateral nucleus‐bilateral inferior occipital lobe and right thalamic central lateral nucleus‐right transverse temporal gyrus FC are interpreted as high‐road‐like thalamic‐sensory‐cortical involvement. We therefore consider that our findings point to abnormalities in both routes in MDD patients.

For the low‐road‐like interpretation, it is worth noting, however, that the central lateral nucleus is included in the intralaminar nucleus, and our interpretation of a low‐road‐like effect is not intended to imply a direct structural thalamic central lateral nucleus‐amygdala tract. Canonical descriptions of a monosynaptic thalamic‐amygdala route are more often linked to sensory thalamic territories rather than the intralaminar central lateral nucleus.^71^ At the same time, it is also important to acknowledge that direct thalamic inputs to the amygdala are well documented for other intralaminar nuclei, particularly the central medial nucleus, which is described as strongly targeting the amygdala compared with the central lateral nucleus.^71^ Accordingly, we interpret the observed central lateral nucleus‐amygdala FC increase as reflecting predominantly functional and/or indirect thalamic‐amygdala coupling within broader intralaminar‐limbic circuitry rather than evidence for a direct anatomical pathway from central lateral nucleus to the amygdala.

For the high‐road‐like interpretation, prior work supports the plausibility of central lateral nucleus involvement in thalamic‐cortical pathways. In particular, Vertes *et al*. summarized evidence indicating that there are fibers spanning from the central lateral nucleus projecting to the occipital cortex,^71^ supporting central lateral nucleus involvement in thalamic‐visual cortical pathways relevant to our central lateral nucleus‐inferior occipital lobe SC finding. In addition, diffusion‐based mapping by Kumar *et al*. reported that the central lateral nucleus shows intrinsic thalamic connections to the temporal thalamic projection site,^100^, ^101^ providing a plausible substrate for interpreting our central lateral nucleus‐transverse temporal gyrus SC finding as thalamic‐temporal network coupling rather than a canonical “auditory relay” pathway.^101^

Of note, our structural findings were selective. We found increased streamline count only for the right central lateral nucleus‐right inferior occipital lobe tract, while other SCs (including the right central lateral nucleus‐right transverse temporal gyrus tract and right central lateral nucleus‐right amygdala subfield tracts) showed no significant group difference. This pattern suggests that the high‐road‐like thalamic‐visual cortical pathway may show detectable macrostructural remodeling, whereas the low‐road‐like central lateral nucleus‐amygdala coupling and the high‐road‐like central lateral nucleus‐temporal coupling may be more prominently expressed at the functional level and/or *via* indirect routing that is less reliably captured by diffusion tractography.

Finally, we observed right‐lateralized FC effects for the central lateral nucleus‐amygdala and central lateral nucleus‐transverse temporal gyrus connections. In contrast, the central lateral nucleus‐inferior occipital lobe FC was bilateral, while the streamline count increase was detected only for the right central lateral nucleus‐right inferior occipital lobe tract. We note that robust central lateral nucleus‐specific hemispheric lateralization is not firmly established. Therefore, we interpret laterality cautiously as an empirical observation. Nevertheless, prior work suggests that the right amygdala can be preferentially engaged in rapid or non‐conscious processing of emotionally salient stimuli,^102^, ^103^, ^104^ which is broadly compatible with the right‐lateralized low‐road‐like functional coupling observed here.

### Integration of structural and FC findings

In the present study, we integrated FC and SC to characterize thalamic subnuclei abnormalities in MDD across modalities. Of note, we observed partial convergence at the group level: the right thalamic central lateral nucleus‐right inferior occipital lobe pathway showed both increased FC and increased SC in MDD, suggesting that this thalamic‐visual cortical circuit may exhibit detectable macrostructural remodeling alongside functional hypercoupling. In contrast, thalamic central lateral nucleus‐amygdala and thalamic central lateral nucleus‐right transverse temporal gyrus showed FC alterations without corresponding SC differences, indicating modality dissociation in limbic and temporal networks.

Importantly, convergence at the group level did not translate into strong structure–function coupling. Specifically, the right thalamic central lateral nucleus‐right inferior occipital lobe FC and SC were not significantly associated. This suggests that macrostructural changes and functional coupling abnormalities may vary relatively independently. Several mechanisms may account for this dissociation, including indirect or polysynaptic functional interactions that are not well captured by point‐to‐point tractography, as well as state‐dependent neuromodulatory influences that can gate thalamic‐cortical communication. Together, these findings highlight that thalamic connectivity alterations in MDD may involve both structurally supported changes in sensory‐cortical pathways and functional reconfiguration that is not necessarily proportional to structural changes.

### Added value of UHF MRI

The present findings highlight several insights that were uniquely enabled, or substantially strengthened, by UHF 7.0 Tesla MRI. First, the increased spatial resolution and contrast allowed us to interrogate thalamic connectivity at the level of individual subnuclei, including the central lateral nucleus. This is important because the central lateral nucleus is small and prone to partial‐volume effects at conventional resolutions. Whole thalamus or coarse parcellations may therefore average across heterogeneous subregions and mask nucleus‐specific alterations. Second, the higher‐resolution resting‐state fMRI facilitated the central lateral nucleus FC analyses at a finer anatomical scale. Beyond the whole amygdala level, we were able to examine FC between the central lateral nucleus and amygdala subregions, which are small and particularly susceptible to partial‐volume effects and registration imprecision at conventional field strengths. This subregional approach revealed specific central lateral nucleus‐amygdala subfield effects that would likely be difficult to detect reliably using standard resolution acquisitions. Third, diffusion MRI with higher spatial resolution enabled point‐to‐point tractography that links the same thalamic subnucleus to specific cortical targets. This anatomical granularity allowed us to test whether the abnormal central lateral nucleus FCs were accompanied by detectable structural differences, and to demonstrate that structural changes were selective rather than ubiquitous. Finally, by combining FC and SC within the same UHF framework, we were able to evaluate cross‐modal convergence and dissociation. Notably, although the central lateral nucleus‐occipital connectivity showed alterations in both modalities at the group level, FC and SC were not significantly associated at the individual level, underscoring that functional reconfiguration in MDD is not necessarily proportional to tractography‐derived structural estimates. Together, these findings based on UHF MRI refine the interpretation of thalamic abnormalities in MDD by localizing effects to specific subnuclei and pathways, and by clarifying which aspects of connectivity appear structurally supported versus predominantly functional.

### Strengths and limitations

We used considerably higher spatial resolution than prior work on the thalamus and its connectivity profiles in MDD, with our fMRI data having approximately sevenfold (1.5^3^ mm^3^ vs 3.0^3^ mm^3^) and our dMRI data having approximately threefold (1.4^3^ mm^3^ vs 2.0^3^ mm^3^) higher resolution. We were also able to include a relatively large number of MDD patients for a UHF MRI study (fMRI: *n* = 47; dMRI: *n* = 53), allowing us to pinpoint novel relationships between MDD diagnosis and clinical characteristics with functional and SC of thalamic subnuclei. Furthermore, we integrated functional and SC, which is quite rare, even in previous standard field strength studies.^23^ Our subnuclear‐specific findings based on UHF resolution contribute to building a broader framework for precision psychiatry, where such high‐resolution biomarkers have potential for personalized applications.^105^, ^106^ However, our 7.0 Tesla diffusion protocol was single‐shell rather than multi‐shell. This choice reflected practical UHF constraints (e.g., the need to keep TE and scan time feasible) and our prioritization of high spatial resolution for small thalamic subnuclei.^107^ Nevertheless, single‐shell diffusion limits more advanced multi‐shell/multi‐tissue modeling and may reduce FOD estimation accuracy and tractography specificity relative to optimized multi‐shell acquisitions. Therefore, diffusion‐based SC findings should be interpreted with this consideration in mind. Importantly, the main SC group effect remained robust in sensitivity analyses across tractography parameter settings and after connectome scaling to account for ROI volume and streamline length biases. Due to the economic burden and time constraints associated with conducting UHF MRI studies, we chose to prioritize our primary target group (MDD) which resulted in a relatively smaller HC sample, potentially limiting the ability to detect more subtle differences between MDD and HC participants. Replication in larger and better‐matched cohorts is needed. In addition, even though no differences in FC and SC measures were found between medicated and non‐medicated MDD patients, this study revealed that medicated MDD patients had increased SC when compared with HC. However, due to the limitations of the sample size and collected information, we cannot exclude potential medication effects. Factors such as medication type, dose, and duration should be considered in future studies to further enrich the interpretability of the effect of medication on the results. The cross‐sectional nature of the study moreover precludes conclusions regarding causality, making it difficult to dissect whether reported connectional anomalies are a cause or consequence of MDD.

## Conclusions

Using 7.0 Tesla UHF MRI to probe thalamic subnuclei, we uncovered previously undetected functional and SC alterations in MDD. Notably, the central lateral nucleus exhibited disrupted links with cortical and subcortical networks involved in sensory integration, emotion regulation, and arousal. These results address a key gap in the field by revealing subnuclear‐level connectivity disturbances that may contribute to MDD symptomatology. Future studies should investigate whether therapeutic interventions normalize these thalamic subnuclei connectivity abnormalities.

## Author contributions

W.L.: data analysis, manuscript writing, manuscript revision; S.L.: data analysis, manuscript revision; J.H.: data analysis, manuscript revision; L.A.M.: manuscript revision; L.Li.: manuscript revision; M.C.: study design, manuscript revision; W.Z.: study design, manuscript revision; D.J.V.: study design, manuscript revision; L.Lu.: supervision; manuscript revision; M.A.: supervision; study design, manuscript revision; G.W.: supervision; study design, manuscript revision.

## Disclosure statement

M.C. is a shareholder of Nico‐lab International Ltd. G.W. has received research support from Philips, Biogen, and Bitbrain for unrelated work. Other authors declare that they have no conflict of interest.

## Supporting information


**Figure S1.** Subfields of the amygdala. AAA, anterior amygdala area; AB, accessory basal nucleus; Ba, basal nucleus; CAT, cortico‐amygdaloid transition area; Ce, central nucleus; Co, cortical nucleus; La, lateral nucleus; Me, medial nucleus; PL, paralaminar nucleus. Reprinted with permission from Springer Publishing: European Archives of Psychiatry and Clinical Neuroscience (W. Liu *et al*., 2025).

**Figure S2.** Another corresponding SCs. (a) The right CL‐right TTG tract. (b) The right CL‐right CAT tract. (c) The right CL‐right AB tract. (d) The right CL‐right Ba tract. AB, accessory basal nucleus; Ba, basal nucleus; CAT, cortico‐amygdaloid transition area; CL, central lateral nucleus; SC, structural connectivity; TTG, transverse temporal gyrus.

**Data S1.** Supporting Information.

## Data Availability

The data that support the findings of this study are available on request from the corresponding author. The data are not publicly available due to privacy or ethical restrictions.

## References

[1] Otte C , Gold SM , Penninx BW *et al*. Major depressive disorder. Nat. Rev. Dis. Primers. 2016; 2: 16065. 27629598 10.1038/nrdp.2016.65

[2] Malhi GS , Mann JJ . Depression. Lancet 2018; 392: 2299–2312. 30396512 10.1016/S0140-6736(18)31948-2

[3] Colizoli O , de Gee JW , van der Zwaag W , Donner TH . Functional magnetic resonance imaging responses during perceptual decision‐making at 3 and 7 T in human cortex, striatum, and brainstem. Hum. Brain Mapp. 2022; 43: 1265–1279. 34816533 10.1002/hbm.25719 PMC8837598

[4] Morris LS , Kundu P , Costi S *et al*. Ultra‐high field MRI reveals mood‐related circuit disturbances in depression: A comparison between 3‐Tesla and 7‐Tesla. Transl. Psychiatry 2019; 9: 94. 30770788 10.1038/s41398-019-0425-6 PMC6377652

[5] Young KA , Holcomb LA , Yazdani U , Hicks PB , German DC . Elevated neuron number in the limbic thalamus in major depression. Am. J. Psychiatry 2004; 161: 1270–1277. 15229061 10.1176/appi.ajp.161.7.1270

[6] Zhang Y , Zhang Y , Ai H *et al*. Microstructural deficits of the thalamus in major depressive disorder. Brain Commun. 2022; 4: fcac236. 36196087 10.1093/braincomms/fcac236 PMC9525011

[7] Nugent AC , Davis RM , Zarate CA , Drevets WC . Reduced thalamic volumes in major depressive disorder. Psychiatry Res. 2013; 213: 179–185. 23850106 10.1016/j.pscychresns.2013.05.004 PMC3756884

[8] Kempton MJ , Salvador Z , Munafò MR *et al*. Structural neuroimaging studies in major depressive disorder. Meta‐analysis and comparison with bipolar disorder. Arch. Gen. Psychiatry 2011; 68: 675–690. 21727252 10.1001/archgenpsychiatry.2011.60

[9] Drevets WC , Savitz J , Trimble M . The subgenual anterior cingulate cortex in mood disorders. CNS Spectr. 2008; 13: 663–681. 18704022 10.1017/s1092852900013754 PMC2729429

[10] Hamilton JP , Chen MC , Gotlib IH . Neural systems approaches to understanding major depressive disorder: An intrinsic functional organization perspective. Neurobiol. Dis. 2013; 52: 4–11. 23477309 10.1016/j.nbd.2012.01.015 PMC3596788

[11] Modha DS , Singh R . Network architecture of the long‐distance pathways in the macaque brain. Proc. Natl. Acad. Sci. U. S. A. 2010; 107: 13485–13490. 20628011 10.1073/pnas.1008054107 PMC2922151

[12] Saalmann YB , Pinsk MA , Wang L , Li X , Kastner S . The pulvinar regulates information transmission between cortical areas based on attention demands. Science 2012; 337: 753–756. 22879517 10.1126/science.1223082 PMC3714098

[13] Gallo S , El‐Gazzar A , Zhutovsky P *et al*. Functional connectivity signatures of major depressive disorder: Machine learning analysis of two multicenter neuroimaging studies. Mol. Psychiatry 2023; 28: 3013–3022. 36792654 10.1038/s41380-023-01977-5 PMC10615764

[14] Kang L , Zhang A , Sun N *et al*. Functional connectivity between the thalamus and the primary somatosensory cortex in major depressive disorder: A resting‐state fMRI study. BMC Psychiatry 2018; 18: 339. 30340472 10.1186/s12888-018-1913-6 PMC6194586

[15] Brown EC , Clark DL , Hassel S , MacQueen G , Ramasubbu R . Thalamocortical connectivity in major depressive disorder. J. Affect. Disord. 2017; 217: 125–131. 28407555 10.1016/j.jad.2017.04.004

[16] Coloigner J , Batail J‐M , Commowick O *et al*. White matter abnormalities in depression: A categorical and phenotypic diffusion MRI study. NeuroImage Clin. 2019; 22: 101710. 30849644 10.1016/j.nicl.2019.101710 PMC6406626

[17] Lai CH , Wu YT . Alterations in white matter micro‐integrity of the superior longitudinal fasciculus and anterior thalamic radiation of young adult patients with depression. Psychol. Med. 2014; 44: 2825–2832. 25065445 10.1017/S0033291714000440

[18] Iglesias JE , Insausti R , Lerma‐Usabiaga G *et al*. A probabilistic atlas of the human thalamic nuclei combining ex vivo MRI and histology. Neuroimage 2018; 183: 314–326. 30121337 10.1016/j.neuroimage.2018.08.012 PMC6215335

[19] Polders DL , Leemans A , Hendrikse J , Donahue MJ , Luijten PR , Hoogduin JM . Signal to noise ratio and uncertainty in diffusion tensor imaging at 1.5, 3.0, and 7.0 Tesla. J. Magn. Reson. Imaging 2011; 33: 1456–1463. 21591016 10.1002/jmri.22554

[20] Strotmann B , Heidemann RM , Anwander A *et al*. High‐resolution MRI and diffusion‐weighted imaging of the human habenula at 7 tesla. J. Magn. Reson. Imaging 2014; 39: 1018–1026. 24259421 10.1002/jmri.24252

[21] Zheng W , Tan X , Liu T *et al*. Individualized thalamic parcellation reveals alterations in shape and microstructure of thalamic nuclei in patients with disorder of consciousness. Cereb. Cortex Commun. 2021; 2: tgab024. 34296169 10.1093/texcom/tgab024 PMC8152869

[22] de Kwaasteniet B , Ruhe E , Caan M *et al*. Relation between structural and functional connectivity in major depressive disorder. Biol. Psychiatry 2013; 74: 40–47. 23399372 10.1016/j.biopsych.2012.12.024

[23] Scheepens DS , van Waarde JA , Lok A , de Vries G , Denys DAJP , van Wingen GA . The link between structural and functional brain abnormalities in depression: A systematic review of multimodal neuroimaging studies. Front. Psychiatry. 2020; 11: 485. 32581868 10.3389/fpsyt.2020.00485 PMC7283615

[24] Liu W , Heij J , Liu S *et al*. Structural connectivity of dopaminergic pathways in major depressive disorder: An ultra‐high resolution 7‐Tesla diffusion MRI study. Eur. Neuropsychopharmacol. 2024; 89: 58–70. 39341085 10.1016/j.euroneuro.2024.07.014

[25] Liu W , Heij J , Liu S *et al*. Structural connectivity of thalamic subnuclei in major depressive disorder: An ultra‐high resolution diffusion MRI study at 7‐Tesla. J. Affect. Disord. 2025; 370: 412–426. 39505018 10.1016/j.jad.2024.11.009

[26] Liu W , Heij J , Liu S *et al*. Hippocampal, thalamic, and amygdala subfield morphology in major depressive disorder: An ultra‐high resolution MRI study at 7‐Tesla. Eur. Arch. Psychiatry Clin. Neurosci. 2025; 275: 1113–1129. 39217211 10.1007/s00406-024-01874-0 PMC12149001

[27] Smeets RMW , Dingemans P . Composite International Diagnostic Interview (CIDI), Version 1.1. World Health Organization, Amsterdam/Geneva, 1993.

[28] Rush AJ , Gullion CM , Basco MR , Jarrett RB , Trivedi MH . The inventory of depressive symptomatology (IDS): Psychometric properties. Psychol. Med. 1996; 26: 477–486. 8733206 10.1017/s0033291700035558

[29] Beck AT , Epstein N , Brown G , Steer RA . An inventory for measuring clinical anxiety: Psychometric properties. J. Consult. Clin. Psychol. 1988; 56: 893–897. 3204199 10.1037//0022-006x.56.6.893

[30] Levine DW , Kaplan RM , Kripke DF , Bowen DJ , Naughton MJ , Shumaker SA . Factor structure and measurement invariance of the Women's Health Initiative Insomnia Rating Scale. Psychol. Assess. 2003; 15: 123–136. 12847773 10.1037/1040-3590.15.2.123

[31] de Graaf R , Bijl RV , Smit F , Vollebergh WAM , Spijker J . Risk factors for 12‐month comorbidity of mood, anxiety, and substance use disorders: Findings from The Netherlands mental health survey and incidence study. Am. J. Psychiatry 2002; 159: 620–629. 11925301 10.1176/appi.ajp.159.4.620

[32] Andersson JLR , Sotiropoulos SN . An integrated approach to correction for off‐resonance effects and subject movement in diffusion MR imaging. Neuroimage 2016; 125: 1063–1078. 26481672 10.1016/j.neuroimage.2015.10.019 PMC4692656

[33] Caan MWA , Bazin P‐L , Marques JP , de Hollander G , Dumoulin SO , van der Zwaag W . MP2RAGEME: T1, T2*, and QSM mapping in one sequence at 7 Tesla. Hum. Brain Mapp. 2019; 40: 1786–1798. 30549128 10.1002/hbm.24490 PMC6590660

[34] Marques JP , Kober T , Krueger G , van der Zwaag W , Van de Moortele P‐F , Gruetter R . MP2RAGE, a self bias‐field corrected sequence for improved segmentation and T1‐mapping at high field. Neuroimage 2010; 49: 1271–1281. 19819338 10.1016/j.neuroimage.2009.10.002

[35] Fischl B . FreeSurfer. Neuroimage 2012; 62: 774–781. 22248573 10.1016/j.neuroimage.2012.01.021 PMC3685476

[36] Saygin ZM , Kliemann D , Iglesias JE *et al*. High‐resolution magnetic resonance imaging reveals nuclei of the human amygdala: Manual segmentation to automatic atlas. Neuroimage 2017; 155: 370–382. 28479476 10.1016/j.neuroimage.2017.04.046 PMC5557007

[37] Iglesias JE , Augustinack JC , Nguyen K *et al*. A computational atlas of the hippocampal formation using ex vivo, ultra‐high resolution MRI: Application to adaptive segmentation of in vivo MRI. Neuroimage 2015; 115: 117–137. 25936807 10.1016/j.neuroimage.2015.04.042 PMC4461537

[38] Esteban O , Markiewicz CJ , Blair RW *et al*. fMRIPrep: A robust preprocessing pipeline for functional MRI. Nat. Methods 2019; 16: 111–116. 30532080 10.1038/s41592-018-0235-4 PMC6319393

[39] Gorgolewski K , Burns C , Madison C *et al*. Nipype: A flexible, lightweight and extensible neuroimaging data processing framework in python. Front. Neuroinform. 2011; 5: 13. 21897815 10.3389/fninf.2011.00013 PMC3159964

[40] Snoek L , Casimiro E , Lindh D , Knapen T . lukassnoek/pybest (Version 0.1). Zenodo. 2024. Available from URL: 10.5281/zenodo.10837110.

[41] Kellner E , Dhital B , Kiselev VG , Reisert M . Gibbs‐ringing artifact removal based on local subvoxel‐shifts. Magn. Reson. Med. 2016; 76: 1574–1581. 26745823 10.1002/mrm.26054

[42] Veraart J , Fieremans E , Novikov DS . Diffusion MRI noise mapping using random matrix theory. Magn. Reson. Med. 2016; 76: 1582–1593. 26599599 10.1002/mrm.26059 PMC4879661

[43] Veraart J , Novikov DS , Christiaens D , Ades‐Aron B , Sijbers J , Fieremans E . Denoising of diffusion MRI using random matrix theory. Neuroimage 2016; 142: 394–406. 27523449 10.1016/j.neuroimage.2016.08.016 PMC5159209

[44] Cordero‐Grande L , Christiaens D , Hutter J , Price AN , Hajnal JV . Complex diffusion‐weighted image estimation via matrix recovery under general noise models. Neuroimage 2019; 200: 391–404. 31226495 10.1016/j.neuroimage.2019.06.039 PMC6711461

[45] Andersson JLR , Skare S , Ashburner J . How to correct susceptibility distortions in spin‐echo echo‐planar images: Application to diffusion tensor imaging. Neuroimage 2003; 20: 870–888. 14568458 10.1016/S1053-8119(03)00336-7

[46] Smith SM , Jenkinson M , Woolrich MW *et al*. Advances in functional and structural MR image analysis and implementation as FSL. Neuroimage 2004; 23: S208–S219. 15501092 10.1016/j.neuroimage.2004.07.051

[47] Tustison NJ , Avants BB , Cook PA *et al*. N4ITK: Improved N3 bias correction. IEEE Trans. Med. Imaging 2010; 29: 1310–1320. 20378467 10.1109/TMI.2010.2046908 PMC3071855

[48] Jenkinson M , Bannister P , Brady M , Smith S . Improved optimization for the robust and accurate linear registration and motion correction of brain images. Neuroimage 2002; 17: 825–841. 12377157 10.1016/s1053-8119(02)91132-8

[49] Jenkinson M , Smith S . A global optimisation method for robust affine registration of brain images. Med. Image Anal. 2001; 5: 143–156. 11516708 10.1016/s1361-8415(01)00036-6

[50] Smith RE , Tournier J‐D , Calamante F , Connelly A . Anatomically‐constrained tractography: Improved diffusion MRI streamlines tractography through effective use of anatomical information. Neuroimage 2012; 62: 1924–1938. 22705374 10.1016/j.neuroimage.2012.06.005

[51] Veraart J , Sijbers J , Sunaert S , Leemans A , Jeurissen B . Weighted linear least squares estimation of diffusion MRI parameters: Strengths, limitations, and pitfalls. Neuroimage 2013; 81: 335–346. 23684865 10.1016/j.neuroimage.2013.05.028

[52] Basser PJ , Mattiello J , LeBihan D . Estimation of the effective self‐diffusion tensor from the NMR spin echo. J. Magn. Reson. B 1994; 103: 247–254. 8019776 10.1006/jmrb.1994.1037

[53] Tournier J‐D , Smith R , Raffelt D *et al*. MRtrix3: A fast, flexible and open software framework for medical image processing and visualisation. Neuroimage 2019; 202: 116137. 31473352 10.1016/j.neuroimage.2019.116137

[54] Jeurissen B , Tournier J‐D , Dhollander T , Connelly A , Sijbers J . Multi‐tissue constrained spherical deconvolution for improved analysis of multi‐shell diffusion MRI data. Neuroimage 2014; 103: 411–426. 25109526 10.1016/j.neuroimage.2014.07.061

[55] Basser PJ , Mattiello J , LeBihan D . MR diffusion tensor spectroscopy and imaging. Biophys. J. 1994; 66: 259–267. 8130344 10.1016/S0006-3495(94)80775-1 PMC1275686

[56] Xu K , Liu Y , Zhan Y , Ren J , Jiang T . BRANT: A versatile and extendable resting‐state fMRI toolkit. Front. Neuroinform. 2018; 12: 52. 30233348 10.3389/fninf.2018.00052 PMC6129764

[57] Tournier J‐D , Calamante F , Connelly A . Improved probabilistic streamlines tractography by 2nd order integration over fibre orientation distributions. Proc. Intl. Soc. Mag. Reson. Med. 2010; 18: 1670.

[58] Fuscà M , Siebenhühner F , Wang SH *et al*. Brain criticality predicts individual levels of inter‐areal synchronization in human electrophysiological data. Nat. Commun. 2023; 14: 4736. 37550300 10.1038/s41467-023-40056-9 PMC10406818

[59] Jellema PEJ , Kersbergen KJ , Hoving EW *et al*. Evaluation of tractography parameters for dentato‐rubro‐thalamic tract reconstruction during pediatric posterior fossa tumor surgery. Magn. Reson. Mater. Phys. Biol. Med. 2025. Available from URL: 10.1007/s10334-025-01297-5. PMC13124821 41026333

[60] Brown SSG , Rutland JW , Verma G *et al*. Ultra‐high‐resolution imaging of amygdala subnuclei structural connectivity in major depressive disorder. Biol. Psychiatry: Cognit. Neurosci. Neuroimaging 2020; 5: 184–193. 31570286 10.1016/j.bpsc.2019.07.010 PMC7010542

[61] Plantinga B , Roebroeck A , Kemper V *et al*. Ultra‐high field MRI post mortem structural connectivity of the human subthalamic nucleus, substantia nigra, and globus pallidus. Front. Neuroanat. 2016; 10: 66. 27378864 10.3389/fnana.2016.00066 PMC4909758

[62] Smith RE , Tournier J‐D , Calamante F , Connelly A . SIFT: Spherical‐deconvolution informed filtering of tractograms. Neuroimage 2013; 67: 298–312. 23238430 10.1016/j.neuroimage.2012.11.049

[63] Smith RE , Tournier J‐D , Calamante F , Connelly A . SIFT2: Enabling dense quantitative assessment of brain white matter connectivity using streamlines tractography. Neuroimage 2015; 119: 338–351. 26163802 10.1016/j.neuroimage.2015.06.092

[64] Derrick B , White P . Why Welch's test is type I error robust. Quant. Methods Psychol. 2016; 12: 30–38.

[65] Delacre M , Lakens D , Leys C . Why psychologists should by default use Welch's t‐test instead of Student's t‐test. Int. Rev. Soc. Psychol. 2017; 30: 92–101.

[66] Wainberg M , Forde NJ , Mansour S *et al*. Genetic architecture of the structural connectome. Nat. Commun. 2024; 15: 1962. 38438384 10.1038/s41467-024-46023-2 PMC10912129

[67] Girard G , Whittingstall K , Deriche R , Descoteaux M . Towards quantitative connectivity analysis: Reducing tractography biases. Neuroimage 2014; 98: 266–278. 24816531 10.1016/j.neuroimage.2014.04.074

[68] Aydogan DB , Jacobs R , Dulawa S *et al*. When tractography meets tracer injections: A systematic study of trends and variation sources of diffusion‐based connectivity. Brain Struct. Funct. 2018; 223: 2841–2858. 29663135 10.1007/s00429-018-1663-8 PMC5997540

[69] Donahue CJ , Sotiropoulos SN , Jbabdi S *et al*. Using diffusion tractography to predict cortical connection strength and distance: A quantitative comparison with tracers in the monkey. J. Neurosci. 2016; 36: 6758–6770. 27335406 10.1523/JNEUROSCI.0493-16.2016 PMC4916250

[70] Hagmann P , Cammoun L , Gigandet X *et al*. Mapping the structural core of human cerebral cortex. PLoS Biol. 2008; 6: e159. 18597554 10.1371/journal.pbio.0060159 PMC2443193

[71] Vertes RP , Linley SB , Rojas AKP . Structural and functional organization of the midline and intralaminar nuclei of the thalamus. Front. Behav. Neurosci. 2022; 16: 964644. 36082310 10.3389/fnbeh.2022.964644 PMC9445584

[72] Schmidt FM , Sander C , Dietz M‐E *et al*. Brain arousal regulation as response predictor for antidepressant therapy in major depression. Sci. Rep. 2017; 7: 45187. 28345662 10.1038/srep45187 PMC5366924

[73] Douglas KM , Porter RJ . Longitudinal assessment of neuropsychological function in major depression. Aust. N. Z. J. Psychiatry 2009; 43: 1105–1117. 20001409 10.3109/00048670903279887

[74] Bora E , Harrison BJ , Yücel M , Pantelis C . Cognitive impairment in euthymic major depressive disorder: A meta‐analysis. Psychol. Med. 2013; 43: 2017–2026. 23098294 10.1017/S0033291712002085

[75] Groves SJ , Douglas KM , Porter RJ . A systematic review of cognitive predictors of treatment outcome in major depression. Front. Psychiatry. 2018; 9: 382. 30210368 10.3389/fpsyt.2018.00382 PMC6121150

[76] Surova G , Ulke C , Schmidt FM , Hensch T , Sander C , Hegerl U . Fatigue and brain arousal in patients with major depressive disorder. Eur. Arch. Psychiatry Clin. Neurosci. 2021; 271: 527–536. 33275166 10.1007/s00406-020-01216-w PMC7981331

[77] Roddy D , Kelly JR , Farrell C *et al*. Amygdala substructure volumes in major depressive disorder. NeuroImage Clin. 2021; 31: 102781. 34384996 10.1016/j.nicl.2021.102781 PMC8361319

[78] Saygin ZM , Osher DE , Augustinack J , Fischl B , Gabrieli JDE . Connectivity‐based segmentation of human amygdala nuclei using probabilistic tractography. Neuroimage 2011; 56: 1353–1361. 21396459 10.1016/j.neuroimage.2011.03.006 PMC3102511

[79] Bach DR , Behrens TE , Garrido L , Weiskopf N , Dolan RJ . Deep and superficial amygdala nuclei projections revealed in vivo by probabilistic tractography. J. Neurosci. 2011; 31: 618–623. 21228170 10.1523/JNEUROSCI.2744-10.2011 PMC3059574

[80] Zhou H‐Y , He J‐G , Hu Z‐L *et al*. A‐kinase anchoring protein 150 and protein kinase A complex in the basolateral amygdala contributes to depressive‐like behaviors induced by chronic restraint stress. Biol. Psychiatry 2019; 86: 131–142. 31076080 10.1016/j.biopsych.2019.03.967

[81] Liu W‐Z , Zhang W‐H , Zheng Z‐H *et al*. Identification of a prefrontal cortex‐to‐amygdala pathway for chronic stress‐induced anxiety. Nat. Commun. 2020; 11: 2221. 32376858 10.1038/s41467-020-15920-7 PMC7203160

[82] Rubinow MJ , Mahajan G , May W *et al*. Basolateral amygdala volume and cell numbers in major depressive disorder: A postmortem stereological study. Brain Struct. Funct. 2016; 221: 171–184. 25287512 10.1007/s00429-014-0900-z PMC4388764

[83] Ho TC , Gutman B , Pozzi E *et al*. Subcortical shape alterations in major depressive disorder: Findings from the ENIGMA major depressive disorder working group. Hum. Brain Mapp. 2022; 43: 341–351. 32198905 10.1002/hbm.24988 PMC8675412

[84] Cong E , Li Q , Chen H *et al*. Association between the volume of subregions of the amygdala and major depression with suicidal thoughts and anxiety in a Chinese cohort. J. Affect. Disord. 2022; 312: 39–45. 35691414 10.1016/j.jad.2022.05.122

[85] Monereo‐Sánchez J , Jansen JFA , Van Boxtel MPJ *et al*. Association of hippocampal subfield volumes with prevalence, course and incidence of depressive symptoms: The Maastricht study. Br. J. Psychiatry 2024; 224: 66–73. 37993980 10.1192/bjp.2023.143 PMC10807974

[86] Yang C , Xiao K , Ao Y , Cui Q , Jing X , Wang Y . The thalamus is the causal hub of intervention in patients with major depressive disorder: Evidence from the Granger causality analysis. Neuroimage Clin. 2022; 37: 103295. 36549233 10.1016/j.nicl.2022.103295 PMC9795532

[87] Price JL , Drevets WC . Neurocircuitry of mood disorders. Neuropsychopharmacology 2010; 35: 192–216. 19693001 10.1038/npp.2009.104 PMC3055427

[88] Zhao D , Liu C , Cui M *et al*. The paraventricular thalamus input to central amygdala controls depression‐related behaviors. Exp. Neurol. 2021; 342: 113744. 33965409 10.1016/j.expneurol.2021.113744

[89] Rehman A , Al Khalili Y . Neuroanatomy, occipital lobe. In: StatPearls. StatPearls Publishing, Treasure Island, FL, 2024. 31335040

[90] Wu F , Lu Q , Kong Y , Zhang Z . A comprehensive overview of the role of visual cortex malfunction in depressive disorders: Opportunities and challenges. Neurosci. Bull. 2023; 39: 1426–1438. 36995569 10.1007/s12264-023-01052-7 PMC10062279

[91] Sanacora G , Gueorguieva R , Epperson CN *et al*. Subtype‐specific alterations of γ‐aminobutyric acid and glutamatein patients with major depression. Arch. Gen. Psychiatry 2004; 61: 705–713. 15237082 10.1001/archpsyc.61.7.705

[92] Fullard K , Maller JJ , Welton T *et al*. Is occipital bending a structural biomarker of risk for depression and sensitivity to treatment? J. Clin. Neurosci. 2019; 63: 55–61. 30827879 10.1016/j.jocn.2019.02.007

[93] Lu F , Cui Q , Huang X *et al*. Anomalous intrinsic connectivity within and between visual and auditory networks in major depressive disorder. Prog. Neuropsychopharmacol. Biol. Psychiatry 2020; 100: 109889. 32067960 10.1016/j.pnpbp.2020.109889

[94] van Dam WO , Chrysikou EG . Effects of unilateral tDCS over left prefrontal cortex on emotion regulation in depression: Evidence from concurrent functional magnetic resonance imaging. Cogn. Affect. Behav. Neurosci. 2021; 21: 14–34. 33432545 10.3758/s13415-020-00830-4 PMC8572372

[95] Kähkönen S , Yamashita H , Rytsälä H , Suominen K , Ahveninen J , Isometsä E . Dysfunction in early auditory processing in major depressive disorder revealed by combined MEG and EEG. J. Psychiatry Neurosci. 2007; 32: 316–322. 17823647 PMC1963351

[96] Liu W , Li H , Lin X *et al*. Blunted superior temporal gyrus activity to negative emotional expression after mindfulness‐based cognitive therapy for late‐life depression. Front. Aging Neurosci. 2022; 14: 1001447. 36329872 10.3389/fnagi.2022.1001447 PMC9623567

[97] LeDoux JE . The Emotional Brain: The Mysterious Underpinnings of Emotional Life. Simon and Schuster, New York, 1998.

[98] Méndez‐Bértolo C , Moratti S , Toledano R *et al*. A fast pathway for fear in human amygdala. Nat. Neurosci. 2016; 19: 1041–1049. 27294508 10.1038/nn.4324

[99] Debiec J , LeDoux J . Fear and the brain. Soc. Res. 2004; 71: 807–818.

[100] Kumar VJ , Scheffler K , Grodd W . The structural connectivity mapping of the intralaminar thalamic nuclei. Sci. Rep. 2023; 13: 11938. 37488187 10.1038/s41598-023-38967-0 PMC10366221

[101] Hwang K , Bertolero MA , Liu WB , D'Esposito M . The human thalamus is an integrative hub for functional brain networks. J. Neurosci. 2017; 37: 5594–5607. 28450543 10.1523/JNEUROSCI.0067-17.2017 PMC5469300

[102] Xie T , van Rooij SJH , Inman CS , Wang S , Brunner P , Willie JT . The case for hemispheric lateralization of the human amygdala in fear processing. Mol. Psychiatry 2025; 30: 2252–2259. 40016388 10.1038/s41380-025-02940-2 PMC12014508

[103] Varkevisser T , Geuze E , van Honk J . Amygdala fMRI – A critical appraisal of the extant literature. Neurosci. Insights 2024; 19: 26331055241270591. 39148643 10.1177/26331055241270591 PMC11325331

[104] Dahlén AD , Schofield A , Schiöth HB , Brooks SJ . Subliminal emotional faces elicit predominantly right‐lateralized amygdala activation: A systematic meta‐analysis of fMRI studies. Front. Neurosci. 2022; 16: 868366. 35924231 10.3389/fnins.2022.868366 PMC9339677

[105] Viessmann O , Polimeni JR . High‐resolution fMRI at 7 Tesla: Challenges, promises and recent developments for individual‐focused fMRI studies. Curr. Opin. Behav. Sci. 2021; 40: 96–104. 33816717 10.1016/j.cobeha.2021.01.011 PMC8018601

[106] Gao Q‐L , Chen X , Castellanos FX , Lu B , Yan C‐G . Towards closed‐loop precision psychiatry: Integrating MRI biomarkers for individualized care of major depressive disorder. Psychoradiology 2025; 5: kkaf024. 40933769 10.1093/psyrad/kkaf024 PMC12418937

[107] Liebrand LC , van Wingen GA , Vos FM , Denys D , Caan MWA . Spatial versus angular resolution for tractography‐assisted planning of deep brain stimulation. NeuroImage Clin. 2020; 25: 102116. 31862608 10.1016/j.nicl.2019.102116 PMC6928456

