# Supplementary figures and images for: Functional and structural connectivity of thalamic subnuclei in major depressive disorder at 7 Tesla
Source: Psychiatry Clin Neurosci. 2026 Mar 11;80(6):477–89. doi: 10.1111/pcn.70048 (PMC13244590; doi:10.1111/pcn.70048)

Sagittal

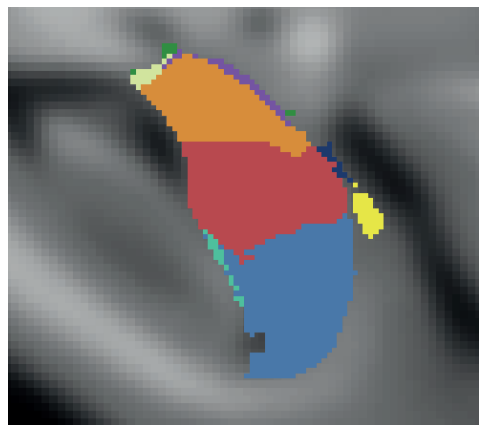

Coronal

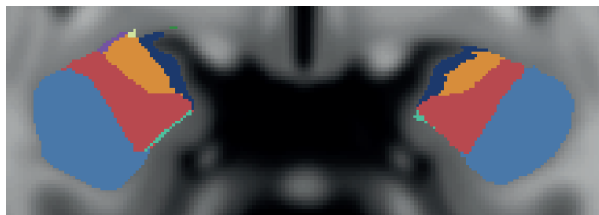

Axial

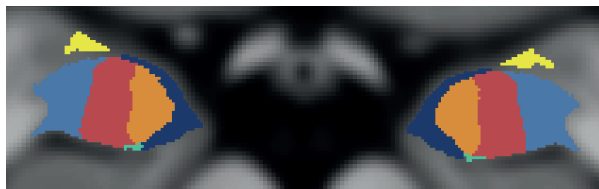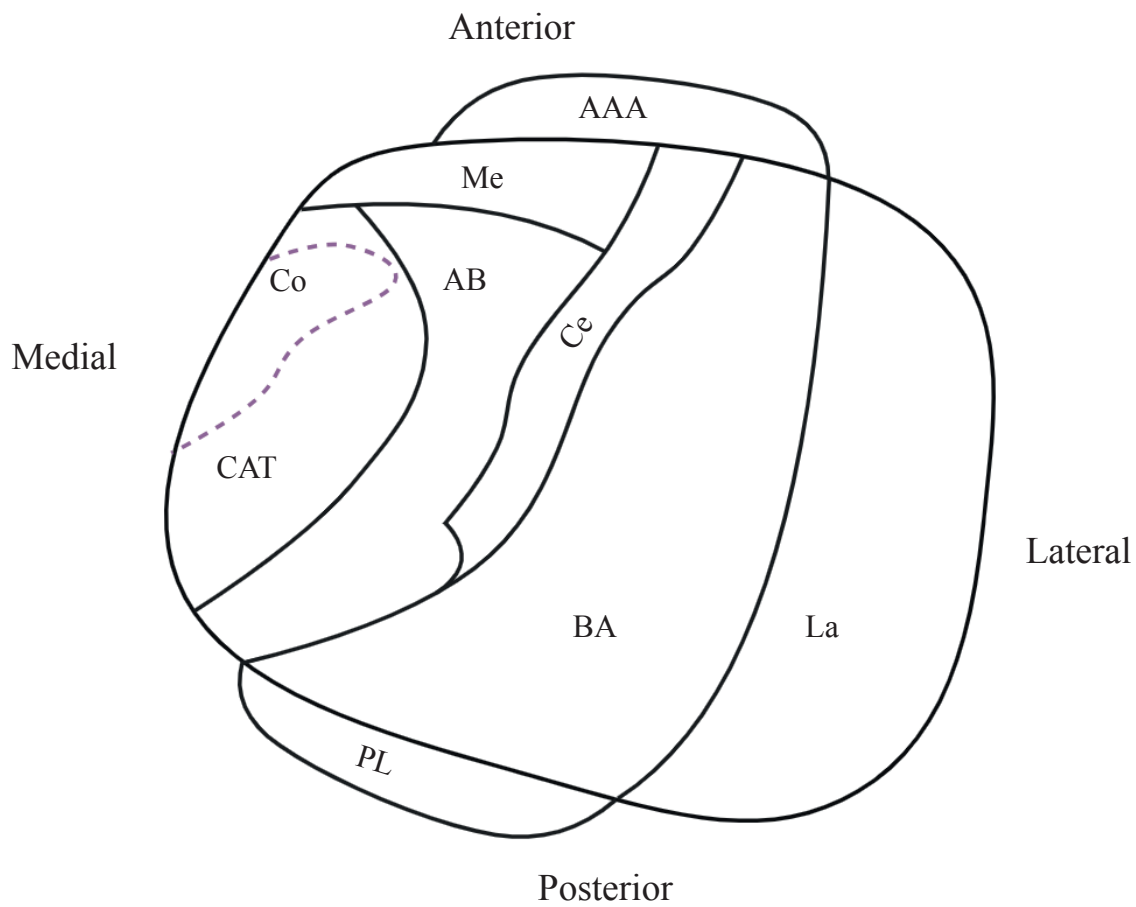

Supplement: Supplementary file 1 — Figure S1. Subfields of the amygdala. AAA, anterior amygdala area; AB, accessory basal nucleus; Ba, basal nucleus; CAT, cortico‐amygdaloid transition area; Ce, central nucleus; Co, cortical nucleus; La, lateral nucleus; Me, medial nucleus; PL, paralaminar nucleus. Reprinted with permission from Springer Publishing: European Archives of Psychiatry and Clinical Neuroscience (W. Liu et al., 2025). [file PCN-80-477-s003.pdf]
