# Supplementary material for: Functional and structural connectivity of thalamic subnuclei in major depressive disorder at 7 Tesla
Source: Psychiatry Clin Neurosci. 2026 Mar 11;80(6):477–89. doi: 10.1111/pcn.70048 (PMC13244590; doi:10.1111/pcn.70048)
Supplement: Supplementary file 2 — Figure S2. Another corresponding SCs. (a) The right CL‐right TTG tract. (b) The right CL‐right CAT tract. (c) The right CL‐right AB tract. (d) The right CL‐right Ba tract. AB, accessory basal nucleus; Ba, basal nucleus; CAT, cortico‐amygdaloid transition area; CL, central lateral nucleus; SC, structural connectivity; TTG, transverse temporal gyrus. [file PCN-80-477-s002.pdf]

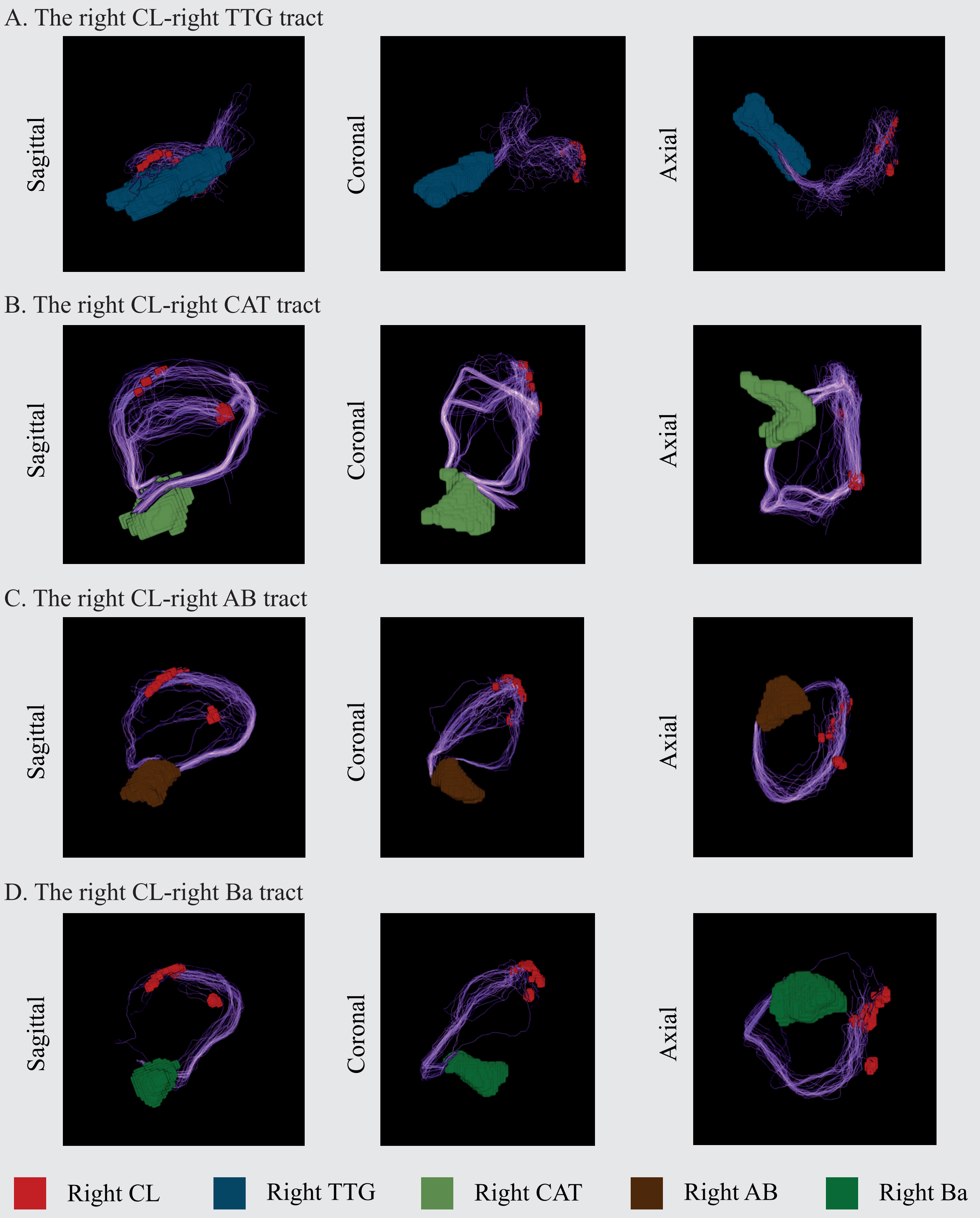
A. The right CL-right TTG tract
Sagital
Coronal
Axial
B. The right CL-right CAT tract
Sagital
Coronal
Axial
C. The right CL-right AB tract
Sagital
Coronal
Axial
D. The right CL-right Ba tract
Sagital
Coronal
Axial
Right CL
Right TTG
Right CAT
Right AB
Right Ba
