# Supplementary material for: Functional and structural connectivity of thalamic subnuclei in major depressive disorder at 7 Tesla
Source: Psychiatry Clin Neurosci. 2026 Mar 11;80(6):477–89. doi: 10.1111/pcn.70048 (PMC13244590; doi:10.1111/pcn.70048)
Supplement: Supplementary file 3 — Data S1. Supporting Information. [file PCN-80-477-s001.docx]

**Supplemental Methods**

***Anatomical data preprocessing***

Each T1-weighted (T1w) image was corrected for intensity non-uniformity (INU) with N4BiasFieldCorrection ^1^, distributed with ANTs 2.2.0 ^2^, and used as T1w-reference throughout the workflow. The T1w-reference was then skull-stripped with a Nipype implementation of the antsBrainExtraction.sh workflow (from ANTs), using OASIS30ANTs as target template. Brain tissue segmentation of cerebrospinal fluid (CSF), white-matter (WM) and gray-matter (GM) was performed on the brain-extracted T1w using fast (FSL 5.0.9) ^3^. Brain surfaces were reconstructed using recon-all (FreeSurfer 6.0.1) ^4^, and the brain mask estimated previously was refined with a custom variation of the method to reconcile ANTs-derived and FreeSurfer-derived segmentations of the cortical gray-matter of Mindboggle ^5^. Volume-based spatial normalization to two standard spaces (MNI152NLin2009cAsym, MNI152NLin6Asym) was performed through nonlinear registration with antsRegistration (ANTs 2.2.0), using brain-extracted versions of both T1w reference and the T1w template. The following templates were selected for spatial normalization: FSL’s MNI ICBM 152 non-linear 6th Generation Asymmetric Average Brain Stereotaxic Registration Model ^6^ [TemplateFlow ID: MNI152NLin6Asym], ICBM 152 Nonlinear Asymmetrical template version 2009c ^7^ [TemplateFlow ID: MNI152NLin2009cAsym].

***Functional data preprocessing***

Resting state data were processed with fMRIPrep version 20.1.1 ^8^ which is based on Nipype 1.5.0 ^9^. First, a reference volume and its skull-stripped version were generated using a custom methodology of fMRIPrep. Head-motion parameters with respect to the BOLD reference (transformation matrices, and six corresponding rotation and translation parameters) are estimated before any spatiotemporal filtering using mcflirt (FSL 5.0.9) ^10^. BOLD runs were slice-time corrected using 3dTshift from AFNI 20160207 ^11^. A fieldmap was estimated based on two echo-planar imaging (EPI) references with opposing phase-encoding directions, with 3dQwarp (AFNI 20160207) ^11^. Based on the estimated susceptibility distortion, a corrected EPI (echo-planar imaging) reference was calculated for a more accurate co-registration with the anatomical reference. The BOLD reference was then co-registered to the T1w reference using bbregister (FreeSurfer) which implements boundary-based registration ^12^. Co-registration was configured with six degrees of freedom. The BOLD time-series (including slice-timing correction when applied) were resampled onto their original, native space by applying a single, composite transform to correct for head-motion and susceptibility distortions. These resampled BOLD time-series will be referred to as preprocessed BOLD in original space, or just preprocessed BOLD. The BOLD time-series were resampled into standard space, generating a preprocessed BOLD run in MNI152NLin6Asym space. First, a reference volume and its skull-stripped version were generated using a custom methodology of fMRIPrep. Several confounding time-series were calculated based on the preprocessed BOLD: framewise displacement (FD), DVARS and three region-wise global signals. FD was computed using two formulations following Power (absolute sum of relative motions) ^13^ and Jenkinson (relative root mean square displacement between affines) ^10^. FD and DVARS are calculated for each functional run, both using their implementations in Nipype (following the definitions by) ^13^. The three global signals are extracted within the CSF, the WM, and the whole-brain masks. Additionally, a set of physiological regressors were extracted to allow for component-based noise correction (CompCor) ^14^. Principal components are estimated after high-pass filtering the preprocessed BOLD time-series (using a discrete cosine filter with 128s cut-off) for the two CompCor variants: temporal (tCompCor) and anatomical (aCompCor). tCompCor components are then calculated from the top 5% variable voxels within a mask covering the subcortical regions. This subcortical mask is obtained by heavily eroding the brain mask, which ensures it does not include cortical GM regions. For aCompCor, components are calculated within the intersection of the aforementioned mask and the union of CSF and WM masks calculated in T1w space, after their projection to the native space of each functional run (using the inverse BOLD-to-T1w transformation). Components are also calculated separately within the WM and CSF masks. For each CompCor decomposition, the k components with the largest singular values are retained, such that the retained components’ time series are sufficient to explain 50 percent of variance across the nuisance mask (CSF, WM, combined, or temporal). The remaining components are dropped from consideration. The head-motion estimates calculated in the correction step were also placed within the corresponding confounds file. The confound time series derived from head motion estimates and global signals were expanded with the inclusion of temporal derivatives and quadratic terms for each ^15^. Frames that exceeded a threshold of 0.5 mm FD or 1.5 standardised DVARS were annotated as motion outliers. All resamplings can be performed with a single interpolation step by composing all the pertinent transformations (i.e. head-motion transform matrices, susceptibility distortion correction when available, and co-registrations to anatomical and output spaces). Gridded (volumetric) resamplings were performed using antsApplyTransforms (ANTs), configured with Lanczos interpolation to minimize the smoothing effects of other kernels ^16^. Non-gridded (surface) resamplings were performed using mri_vol2surf (FreeSurfer).

Many internal operations of fMRIPrep use Nilearn 0.6.2 ^17^, mostly within the functional processing workflow. For more details of the pipeline, see the section corresponding to workflows in fMRIPrep’s documentation.

Following noise regressor estimation, we regressed out all regressors using pybest ^18^. All noise regressors were decomposed into 10 principal components, which were then removed voxel-wise from the timecourses. Principal component removal was done using5-fold cross-validation to optimize hyperparameter settings on the number of principal components to remove per voxel.

**Reference:**

1. Tustison NJ, Avants BB, Cook PA, Zheng Y, Egan A, Yushkevich PA, et al. N4ITK: improved N3 bias correction. *IEEE Trans. Med. Imaging*. 2010; 29: 1310–20.

2. Avants BB, Epstein CL, Grossman M, Gee JC. Symmetric Diffeomorphic Image Registration with Cross-Correlation: Evaluating Automated Labeling of Elderly and Neurodegenerative Brain. *Med. Image Anal.* 2008; 12: 26–41.

3. Zhang Y, Brady M, Smith S. Segmentation of brain MR images through a hidden Markov random field model and the expectation-maximization algorithm. *IEEE Trans. Med. Imaging*. 2001; 20: 45–57.

4. Dale AM, Fischl B, Sereno MI. Cortical Surface-Based Analysis: I. Segmentation and Surface Reconstruction. *NeuroImage*. 1999; 9: 179–94.

5. Klein A, Ghosh SS, Bao FS, Giard J, Häme Y, Stavsky E, et al. Mindboggling morphometry of human brains. *PLoS Comput. Biol.* 2017; 13: e1005350.

6. Evans AC, Janke AL, Collins DL, Baillet S. Brain templates and atlases. *NeuroImage*. 2012; 62: 911–22.

7. Fonov V, Evans A, Mckinstry R, Almli CR, Collins L. Unbiased nonlinear average age-appropriate brain templates from birth to adulthood. *Neuroimage*. 2009; 47.

8. Esteban O, Markiewicz CJ, Blair RW, Moodie CA, Isik AI, Erramuzpe A, et al. fMRIPrep: a robust preprocessing pipeline for functional MRI. *Nat. Methods*. 2019; 16: 111–6.

9. Gorgolewski K, Burns C, Madison C, Clark D, Halchenko Y, Waskom M, et al. Nipype: A Flexible, Lightweight and Extensible Neuroimaging Data Processing Framework in Python. *Front. Neuroinformatics*. 2011; 5:13.

10. Jenkinson M, Bannister P, Brady M, Smith S. Improved optimization for the robust and accurate linear registration and motion correction of brain images. *NeuroImage*. 2002; 17: 825–41.

11. Cox RW, Hyde JS. Software tools for analysis and visualization of fMRI data. *NMR Biomed.* 1997; 10: 171–8.

12. Greve DN, Fischl B. Accurate and robust brain image alignment using boundary-based registration. *NeuroImage*. 2009; 48: 63–72.

13. Power JD, Mitra A, Laumann TO, Snyder AZ, Schlaggar BL, Petersen SE. Methods to detect, characterize, and remove motion artifact in resting state fMRI. *NeuroImage*. 2014; 84: 320–41.

14. Behzadi Y, Restom K, Liau J, Liu TT. A component based noise correction method (CompCor) for BOLD and perfusion based fMRI. *NeuroImage*. 2007; 37: 90–101.

15. Satterthwaite TD, Elliott MA, Gerraty RT, Ruparel K, Loughead J, Calkins ME, et al. An improved framework for confound regression and filtering for control of motion artifact in the preprocessing of resting-state functional connectivity data. *NeuroImage*. 2013; 64: 240–56.

16. Lanczos C. Evaluation of Noisy Data. *J. Soc. Ind. Appl. Math. Ser. B Numer. Anal.* 1964; 1: 76–85.

17. Abraham A, Pedregosa F, Eickenberg M, Gervais P, Mueller A, Kossaifi J, et al. Machine learning for neuroimaging with scikit-learn. *Front. Neuroinformatics*. 2014; 8.

18. Snoek L, ecasimiro, Lindh D, Knapen T. lukassnoek/pybest (Version 0.1). Zenodo. 2024; doi: 10.5281/zenodo.10837110.

| **Supplemental Table 1. Demographical and clinical information of the participants in dMRI analysis.** | | | | | | |
| --- | --- | --- | --- | --- | --- | --- |
|  | | **MDD (n = 53)**  **(n = 53)** | | **HC (n = 12)** | ***t/*** ***χ²*** | ***P*** |
| Age (years), mean ± SD |  | 36.57 ± 10.71 |  | 34.37 ± 9.67 | -0.70 | 0.495 |
| Gender (female), n (%) |  | 40 (75.47%) |  | 7 (58.33%) | 1.44 | 0.231 |
| Age of onset (years) ^a^, mean ± SD |  | 21.33 ± 10.50 |  | - |  | - |
| Recurrent depression, n (%) |  | 22 (41.51%) |  | - |  | - |
| Comorbid anxiety disorder, n (%) |  | 23 (43.40%) |  | - |  | - |
| IDS ^a^, mean ± SD |  | 34.21 ± 13.19 |  | 4.08 ± 2.81 | **-15.04** | **< 0.001** |
| BAI ^a^, mean ± SD |  | 14.33 ± 9.67 |  | 2.42 ± 2.35 | **-7.92** | **< 0.001** |
| IRS ^a^, mean ± SD |  | 9.81 ± 5.16 |  | 5.25 ± 4.43 | **-3.11** | **0.006** |
| CTQ ^a^, mean ± SD |  | 47.15 ± 17.78 |  | 35.58 ± 6.78 | **-3.68** | **< 0.001** |
| With any psychotropic medications, n (%) |  | 30 (56.60%) |  | - |  | - |
| With any antidepressants, n (%) |  | 27 (50.94%) |  | - |  | - |
| With SSRIs and/or SNRIs, n (%) |  | 20 (37.74%) |  | - |  | - |
| With TCA, n (%) |  | 8 (15.09%) |  | - |  | - |
| With atypical antidepressants, n (%) |  | 2 (3.77%) |  | - |  | - |
| With lithium stabilizer, n (%) |  | 3 (5.66%) |  | - |  | - |
| With antipsychotics, n (%) |  | 6 (11.32%) |  | - |  | - |
| With benzodiazepines, n (%) |  | 6 (11.32%) |  | - |  | - |
|  |  |  |  |  |  |  |
| Significance: *P* < 0.05.  Bold indicates significant *P* value.  Abbreviation: MDD = major depressive disorder; HC = healthy control; SD = standard deviation; IDS = Inventory for Depressive Symptomatology; BAI = Beck Anxiety Inventory; IRS = Insomnia Rating Scale; CTQ = Childhood Trauma Questionnaire; SSRI = selective serotonin reuptake inhibitor; SNRI = serotonin-norepinephrine reuptake inhibitor; TCA = tricyclic antidepressant.  ^a^: One MDD patient did not provide the age of onset, and another MDD patient did not provide data for IDS, BAI, IRS, and CTQ. Therefore, when analyzing the data related to these indicators, the sample size for MDD is 52 individuals.  Note: In the MDD group, one patient was excluded because of a low quality T1 image, one patient for low quality dMRI images, and two patients for lacking dMRI images. In the HC group, one participant was excluded here for low quality dMRI images and one participant for lacking dMRI images. The final sample consisted of 53 participants with a primary diagnosis of MDD and 12 HC participants. | | | | | | |

| **Supplemental Table 2. Sensitivity analyses of the tractography parameters.** | | | | | | | | |
| --- | --- | --- | --- | --- | --- | --- | --- | --- |
| **FOD amplitude cutoff** | **Seeding density** | **Step size** | **Maximum curvature** | ***t*** | ***df*** | ***P_FDR_*** | **95% CI** | ***Cohen’s d*** |
| 0.015 | 500,000 | 0.5 | 60° | -3.53 | 62.96 | **0.002** | [-135.39, -37.56] | -0.74 |
| 0.005 | 500,000 | 0.5 | 60° | -3.21 | 62.98 | **0.002** | [-131.28, -30.60] | -0.67 |
| 0.01 | 400,000 | 0.5 | 60° | -3.54 | 62.84 | **0.002** | [-110.93, -30.85] | -0.73 |
| 0.01 | 600,000 | 0.5 | 60° | -3.49 | 62.86 | **0.002** | [-162.63, -44.26] | -0.72 |
| 0.01 | 500,000 | 0.4 | 60° | -3.35 | 62.40 | **0.002** | [-129.86, -32.86] | -0.69 |
| 0.01 | 500,000 | 0.6 | 60° | -3.37 | 63.00 | **0.002** | [-132.89, -33.99] | -0.71 |
| 0.01 | 500,000 | 0.5 | 45° | -3.55 | 62.77 | **0.002** | [-129.91, -36.35] | -0.75 |
| 0.01 | 500,000 | 0.5 | 75° | -3.13 | 62.87 | **0.003** | [-112.74, -24.95] | -0.66 |
| Significance: *P_FDR_* < 0.05.  Bold indicates significant *P_FDR_* value.  Group differences were tested using Welch’s t-test (two-sided) with age, gender, and ICV as covariates.  FDR correction was applied across eight comparisons (eight sets of tractography parameters).  Abbreviation: FOD = fiber orientation distributions. | | | | | | | | |

| **Supplemental Table 3. Demographical and clinical information of the medicated and non-medicated MDD participants in the SC analysis.** | | | | | | |
| --- | --- | --- | --- | --- | --- | --- |
|  | | **Non-medicated MDD**  **(n = 23)** | **Medicated  MDD**  **(n = 30)** | | ***t/*** ***χ²*** | ***P*** |
| Age (years), mean ± SD |  | 36.13 ± 11.16 |  | 36.91 ± 10.53 | -0.26 | 0.796 |
| Gender (female), n (%) |  | 16 (69.57%) |  | 24 (80.00%) | 0.77 | 0.382 |
| Age of onset (years) ^a^, mean ± SD |  | 22.55 ± 11.60 |  | 20.43 ± 9.72 | 0.71 | 0.479 |
| Recurrent depression, n (%) |  | 10 (43.48%) |  | 12 (40.00%) | 0.07 | 0.799 |
| Comorbid anxiety disorder, n (%) |  | 6 (26.09%) |  | 17 (56.67%) | **4.45** | **0.035** |
| IDS ^a^, mean ± SD |  | 28.82 ± 13.34 |  | 38.17 ± 11.79 | **-2.62** | **0.012** |
| CTQ ^a^, mean ± SD |  | 43.77 ± 14.58 |  | 49.63 ± 19.67 | -1.23 | 0.223 |
| BAI ^a^, mean ± SD |  | 9.23 ± 5.94 |  | 18.07 ± 10.24 | **-3.92** | **< 0.001** |
| IRS ^a^, mean ± SD |  | 9.46 ± 5.41 |  | 10.07 ± 5.04 | -0.42 | 0.680 |
| Significance: *P* < 0.05.  Bold indicates significant *P* value.  Group differences were tested with Welch’s t-test.  Abbreviation: MDD = major depressive disorder; HC = healthy control; SD = standard deviation; IDS = Inventory for Depressive Symptomatology; BAI = Beck Anxiety Inventory; IRS = Insomnia Rating Scale; CTQ = Childhood Trauma Questionnaire.  ^a^: One non-medicated MDD patient did not provide the age of onset, and another non-medicated MDD patient did not provide data for IDS, BAI, IRS, and CTQ. Therefore, when analyzing the data related to these indicators, the sample size for MDD is 22 individuals. | | | | | | |
